# Tumor-derived DNA from pleural effusion supernatant as a promising alternative to tumor tissue in genomic profiling of advanced lung cancer

**DOI:** 10.7150/thno.34070

**Published:** 2019-07-28

**Authors:** Lin Tong, Ning Ding, Xiaoling Tong, Jiamin Li, Yong Zhang, Xiaodan Wang, Xiaobo Xu, Maosong Ye, Chun Li, Xue Wu, Hairong Bao, Xin Zhang, Qunying Hong, Yuanlin Song, Yang W. Shao, Chunxue Bai, Jian Zhou, Jie Hu

**Affiliations:** 1 Department of Pulmonary Medicine, Zhongshan Hospital, Fudan University, Shanghai, 200032, China; 2 Shanghai Respiratory Research Institute, Shanghai, 200032, China; 3 Translational Medicine Research Institute, Geneseeq Technology Inc., Toronto, Ontario, M5G 1L7, Canada; 4 Medical Department, Nanjing Geneseeq Technology Inc., Nanjing, Jiangsu, 210032, China; 5 School of Public Health, Nanjing Medical University, Nanjing, Jiangsu, 210029, China

**Keywords:** lung cancer, pleural effusion, cell free DNA, genomic profiling, next-generation sequencing

## Abstract

Pleural effusion (PE) is commonly observed in advanced lung cancer and was suggested to contain both cell-free tumor DNA and tumor cells. Molecular profiling of PE represents a minimally invasive approach of detecting tumor driver mutations for clinical decision making, especially when tumor tissues are not available. The objective of this study is to investigate the efficacy and precision of detecting gene alterations in PE samples to address the feasibility in clinical use.

**Methods:** Sixty-three metastatic lung cancer patients with (n=30, cohort 1) or without (n=33, cohort 2) matched tumor tissues were enrolled in this study. PE and plasma samples of each patient were collected simultaneously. Supernatant and cell precipitate of PE were processed separately to extract cfDNA (PE-cfDNA) and sediment DNA (sDNA). All samples were subjected to targeted next-generation sequencing (NGS) of 416 cancer-related genes.

**Results:** PE supernatants contain more abundant tumor DNA than PE sediments and plasma samples, suggested by higher mutant allele frequencies (MAF) and elevated mutation detection rate in PE-cfDNA (98.4% vs. 90.5% in PE sDNA vs. 87% in plasma cfDNA). In Cohort 1 with matched tumor tissue, tumor mutational burden (TMB) of PE-cfDNA was similar as tumor tissues (6.4 vs. 5.6), but significantly higher than PE sDNA (median TMB: 3.3) and plasma cfDNA (median TMB: 3.4). Ninety-three percent (27 out of 29) of tissue-determined driver mutations were detected in PE-cfDNA, including alterations in *ALK*, *BRAF, EGFR*, *ERBB2*, *KRAS*, *NF1, PIK3CA*, and *RET*, while only 62% were captured in plasma cfDNA. PE-cfDNA also has the highest detection rate of *EGFR* driver mutations in the full cohort (71% vs. 68% in PE sDNA vs. 59% in plasma cfDNA). Mutation detection from cytological negative and hemorrhagic PE is challenging. Comparatively, PE-cfDNA demonstrated absolute superiority than PE sDNA in such a scenario, suggesting that it is an independent source of tumor DNA and therefore less influenced by the abundance of tumor cells.

**Conclusion:** Genomic profiling of PE-cfDNA offers an alternative, and potentially more meticulous approach in assessing tumor genomics in advanced lung cancer when tumor tissue is not available. Our data further demonstrate that in hemorrhagic or cytologically negative PE samples, PE-cfDNA has higher mutation detection sensitivity than sDNA and plasma cfDNA, and therefore is a more reliable source for genetic testing.

## Introduction

Molecular targeted therapies against driver mutations in patients with non-small cell lung cancer (NSCLC) are already improving patients' survival over traditional chemotherapy 1-6, making it increasingly important to incorporate molecular genetic testing into standard clinical care. However, obtaining tumor tissues for molecular profiling is often difficult and sometimes poses risks to patients. Thus, tumor-derived cell-free DNA (cfDNA) from body fluids, including plasma, pleural effusions (PE), cerebrospinal fluids, urine, and saliva, are being investigated for their feasibility in cancer genomic profiling since they are minimally invasive and potentially superior at representing intra- and inter-tumor heterogeneity than single tumor biopsies 7-11. Plasma cfDNA is the most commonly used liquid biopsy for genetic testing in multiple cancers; however, tumor cfDNA present in the total plasma cfDNA can be as low as ~0.01% 12, making the detection of such low allele frequency mutations very challenging 13.

Advanced lung cancer patients develop PE as a result of impaired pleural fluid drainage and increased plasma extravasation to the pleural space following tumor cell invasion of the pleural cavity 14, 15. While applying thoracentesis for diagnosis and therapeutic purposes, PE can be collected at the same time to avoid additional invasive sampling. PE contains floating malignant cells as well as tumor cfDNA in the supernatant 16. Previous studies were able to detect *EGFR* mutations in the cellular sediments and/or supernatants of PE from lung cancer patients using Sanger sequencing, ARMS PCR, mutant-specific PCR, digital PCR, and proton-based next-generation sequencing (NGS) 17-22. However, the feasibility of using PE for large scale genomic profiling of cancer-relevant genes has not been fully investigated.

In this study, we collected PE and plasma samples simultaneously from 63 patients with advanced lung cancer. Thirty patients also had matched tumor tissues available. PE samples were separated into supernatant and cell sediment fractions for independent extraction of cfDNA (PE-cfDNA) and sediment cell genomic DNA (sDNA). Plasma cfDNA was also prepared. All samples were analyzed using target NGS of 416 cancer-relevant genes for comprehensive genomic profiling. The mutation spectra of matched samples from the same patient were compared to systematically evaluate the concordance of genomic profiles from different sample types, and evaluate the feasibility of using PE-derived DNA for clinical molecular diagnoses.

## Materials and Methods

### Patient recruitment and sample collection

Between February 2016 and May 2017, 63 lung cancer patients with pleural effusion were prospectively enrolled at Zhongshan Hospital, Fudan University (Shanghai, China). The study was approved by the ethics committee of Zhongshan Hospital, Fudan University, and all patients provided informed written consent. All samples were tested in a clinical genomic testing facility (Nanjing Geneseeq Technology Inc., Nanjing, China) with protocols approved by the ethics committee of Zhongshan Hospital, Fudan University.

From each patient, 8-10ml of peripheral blood was collected in EDTA-coated tubes (BD) and centrifuged at 1800g for 10 min within 2h of collection to separate the plasma and white blood cells. Plasma was isolated for the extraction of circulating cell-free DNA (cfDNA) and white blood cell sediments were used for genomic DNA extraction as the germline controls. Formalin-fixed, paraffin-embedded (FFPE) sections of matched tumor tissues from 30 of the 63 patients were obtained and the tumor cell content of each sample was determined by a pathologist. 3-6ml PE aliquots were centrifuged at 2500g for 15 min to separate supernatants from floating cells. PE supernatant was isolated for the extraction of cfDNA and cell sediments were used for genomic DNA extraction as the sDNA. For histological assessments of tumor cells in PE, cell smears were prepared from another aliquoted sediment, and stained with hematoxylin and eosin. The cell content of each smear was observed by a pathologist under a microscope at 400× magnification.

### DNA extraction, library preparation and target enrichment

cfDNA from plasma and PE supernatants was purified using the Circulating Nucleic Acid Kit (Qiagen, Hilden, Germany) following the manufacturer's protocol. Genomic DNA from the PE cell pellets, and white blood cells were extracted using the DNeasy Blood & Tissue Kit (Qiagen), while FFPE genomic DNA was purified using the QIAamp DNA FFPE Tissue Kit (Qiagen). Genomic DNA was qualified using a Nanodrop2000 (Thermo Fisher Scientific, Waltham, MA), and cfDNA fragment distribution was analyzed on a Bioanalyzer 2100 using the High Sensitivity DNA Kit (Agilent Technologies, Santa Clara, CA). All DNA was quantified using the dsDNA HS Assay Kit on a Qubit 3.0 Fluorometer (Life Technologies, Carlsbad, CA).

Sequencing libraries were prepared using the KAPA Hyper Prep Kit (KAPA Biosystems, Wilmington, MA), as described previously 23. Indexed DNA libraries were pooled to up to 2μg of total input. Probe-based hybridization capture of the targeted gene regions covering 416 cancer-related genes (Table S1) was performed as previously described 23.

### Sequencing and data processing

Target enriched libraries were sequenced on the HiSeq4000 platform (Illumina) with 2×150bp pair-end reads. Sequencing data were demultiplexed by bcl2fastq (v2.19), analyzed by Trimmomatic 24 to remove low-quality (quality<15) or N bases, and mapped to the reference hg19 genome (Human Genome version 19) using the Burrows-Wheeler Aligner 25. PCR duplicates were removed by Picard (available at: https://broadinstitute.github.io/picard/). The Genome Analysis Toolkit (GATK) 26 was used to perform local realignments around indels and base quality reassurance. SNPs and indels were called by VarScan2 27 and HaplotypeCaller/UnifiedGenotyper in GATK, with the mutant allele frequency (MAF) cutoff as 0.5% for tissue samples, 0.1% for liquid biopsy samples, and a minimum of three unique mutant reads. Common variants were removed using dbSNP and the 1000 Genome project. Germline mutations were filtered out by comparing to patient's whole blood controls. The resulting somatic variants were further filtered through an in-house list of recurrent sequencing errors that was generated from over 10,000 normal control samples on the same sequencing platform.

Gene fusions were identified by FACTERA 28 and copy number variations (CNVs) were analyzed with ADTEx 29. The log2 ratio cut-off for copy number gain was defined as 2.0 for tissue samples and 1.6 for liquid biopsy samples. A log2 ratio cut-off of 0.67 was used for copy number loss detection in all sample types. The thresholds were determined from previous assay validation using the absolute CNVs detected by droplet digital PCR (ddPCR). Allele-specific CNVs were analyzed by FACETS 30 with a 0.2 drift cut-off for unstable joint segments. The proportion of chromosomal instability (CIN) was calculated by dividing the size of drifted segments by the total segment size. Tumor mutational burden (TMB) in this study was defined as the number of somatic synonymous mutations per megabase in each sample, with hotspot/fusion mutations excluded.

## Results

### Study design and patient demographics

This study enrolled 63 patients (55.6% of male) with stage IV lung cancer who had PE at diagnosis or during disease progression (Table S2 and S3). The median age at enrollment was 64 (range, 34-96) and 68.3% were non-smokers. Thirty-five patients were identified without distant metastasis (M1a stage, 55.6%), seven had a single distant metastasis (M1b stage, 11.1%), and 21 had multiple distant metastases (M1c stage, 33.3%). Histological classifications included 58 adenocarcinomas (92.1%), two adeno-squamous carcinomas (3.2%), and three small cell lung cancers (4.8%). PE and matched plasma samples from all patients were collected simultaneously, while matched tumor tissues were available for 30 patients (cohort 1) (Figure S1). Targeted NGS of 416 cancer-relevant genes was applied to all patients' samples for genomic profiling. The detailed list of somatic alterations identified in each sample of the patients is provided in Table S4.

### Tumor cfDNA is more enriched in PE than in plasma

**Figure 1:**
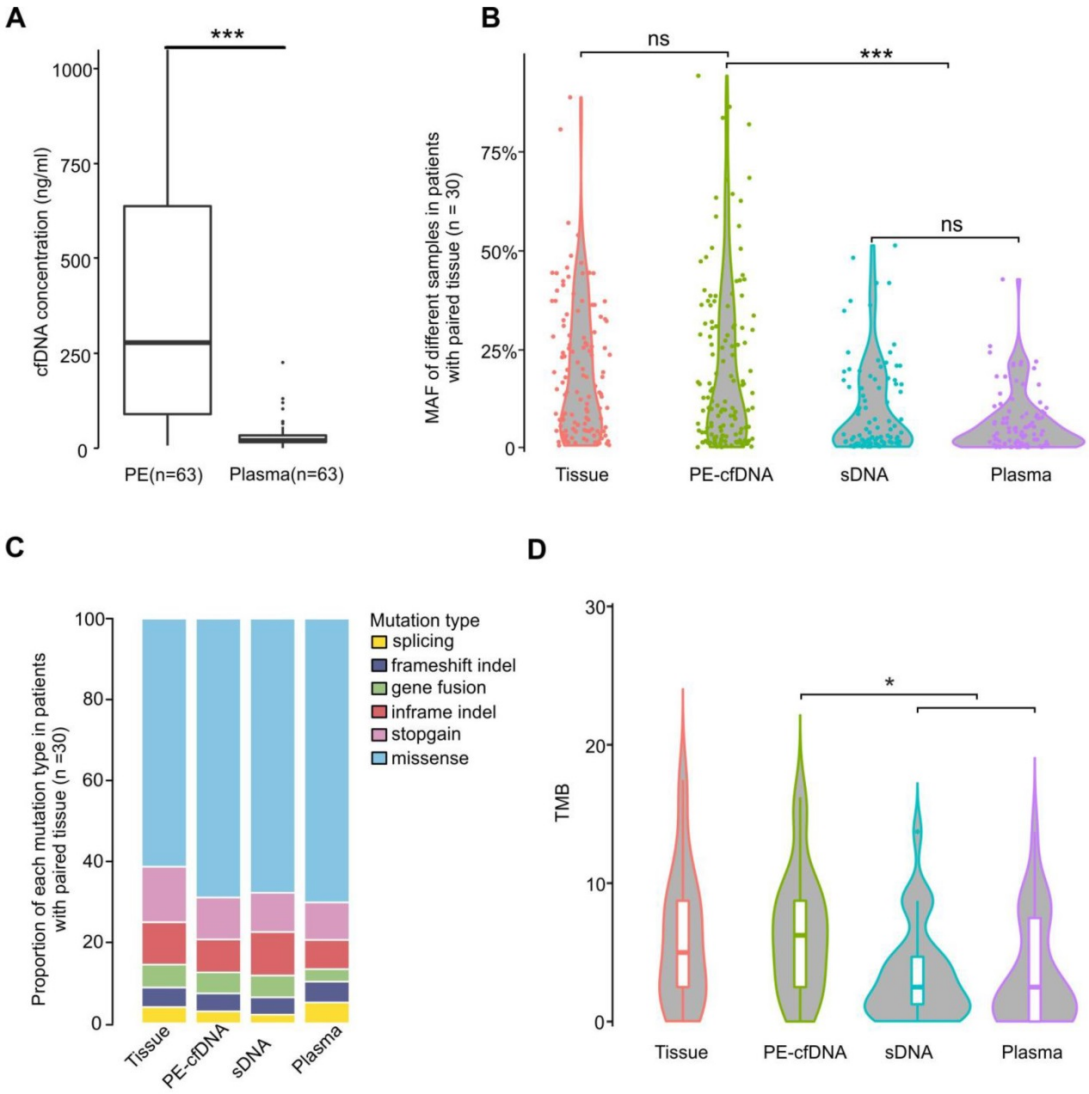
** Characteristics of different sample types.** A) cfDNA concentrations in PE-cfDNA and plasma from all 63 patients. The Mann-Whitney test was used to test the differences between PE-cfDNA and plasma. B) The MAFs of all detected mutations in different samples from cohort 1 patients (n = 30). Each dot represents one patient. C) The proportions of different mutations in each sample type in cohort 1 patients (n = 30). D) TMB distributions in each sample type. For B) and D), the One-way ANOVA on ranks test was used to compare all groups and the Dunn's test was used for post-hoc analyses to compare matched groups. ns, not significant. *, p < 0.05, ***, p < 0.001.

We first observed that total cfDNA concentrations in PE supernatants (median: 278.1ng/ml PE) were much higher than those in plasma (median: 20.4ng/ml plasma) (Figure 1A, p<0.001). Additionally, the mutant allele frequencies (MAFs) of PE-cfDNA were comparable to those identified in the matched tissue samples (Figure 1B), but significantly higher than those of PE sDNA and plasma cfDNA (Figure 1B and S2A), suggesting that the tumor cfDNA fraction in PE-cfDNA was much higher than in sDNA and plasma. Of all patients, 98.4% (62/63) of PE-cfDNA samples have detectable somatic alterations compared to 90.5% (57/63) in PE sDNA and 87% (55/63) in plasma cfDNA (Figure S2B). All genomic alteration types, including single nucleotide variants (SNVs), indels, and gene fusions were detected in similar proportions in each sample type (Figure 1C). The TMBs calculated from PE-cfDNA (median: 6.4) were similar to that of tumor tissues (median: 5.6), but significantly higher than that of sDNA (median: 3.3) and plasma (median: 3.4) (Figure 1D, p<0.05).

Detection of copy number variations (CNVs) in liquid biopsy is always challenging due to its low tumor DNA content. However, PE-cfDNA was also superior in identifying focal CNVs compared to other liquid biopsies. Of 64 CNVs identified in tumor tissues, 20 (31%) were identified in PE-cfDNA, while only 9 (14%) were identified in sDNA, and 3 (5%) were identified in plasma cfDNA (Figure S3A, Table 1). Chromosomal instability (CIN) is another biomarker that relates to disease metastasis, prognosis and treatment resistance^31^. Again, the CIN scores were similar between tumor tissues and PE-cfDNAs, but largely underestimated in sDNA and plasma samples (Figure S3B). Samples from Cohort 2 demonstrated similar results, in which PE-cfDNAs exhibit much better detection rate of CNV and CIN (Figure S4A and S4B).

### PE-cfDNA outperforms other liquid biopsies in detecting tissue-determined mutations and better represents tumor heterogeneity in lung cancer patients

In M1a stage disease, tumors are spatially restricted to the chest, while in M1b/M1c stage disease, one or more distant metastases are present. As a result, PE-cfDNAs demonstrated the highest sensitivity of detecting tissue-determined mutations in M1a disease and had similar sensitivities to sDNA in M1b/M1c disease (Table 2, Figure S5A and S5B) in cohort 1. On the other hand, all liquid biopsy samples contained unique variants that were not detected in matched tumor tissues, especially in M1b/M1c disease with distant metastases. PE-cfDNAs harbored the most unique mutations among different liquid biopsies (Figure S5A and S5C), suggesting that PE-cfDNA was more representative of tumor heterogeneity than sDNA and plasma cfDNA.

For patients lacking matched tumor tissues for NGS test in cohort 2, the liquid biopsy results were compared to the initial amplification-refractory mutation system (ARMS) test of *EGFR* 19del and L858R mutations on diagnostic samples. Of the 24 tumors that underwent the ARMS test, 17 were identified as *EGFR* 19del(+) or L858R(+), and NGS tests of liquid biopsies achieved a sensitivity of 100% with PE-cfDNA, and 88% with sDNA or plasma samples (Figure S6A and S6B). Moreover, one ARMS-classified *EGFR*(-) tumor was reported as *EGFR*(+) by NGS in all liquid biopsy samples (Figure S6A), suggesting a true call by NGS method.

### PE-cfDNA outperforms other liquid biopsy samples for the detection of driver mutations

**Figure 2:**
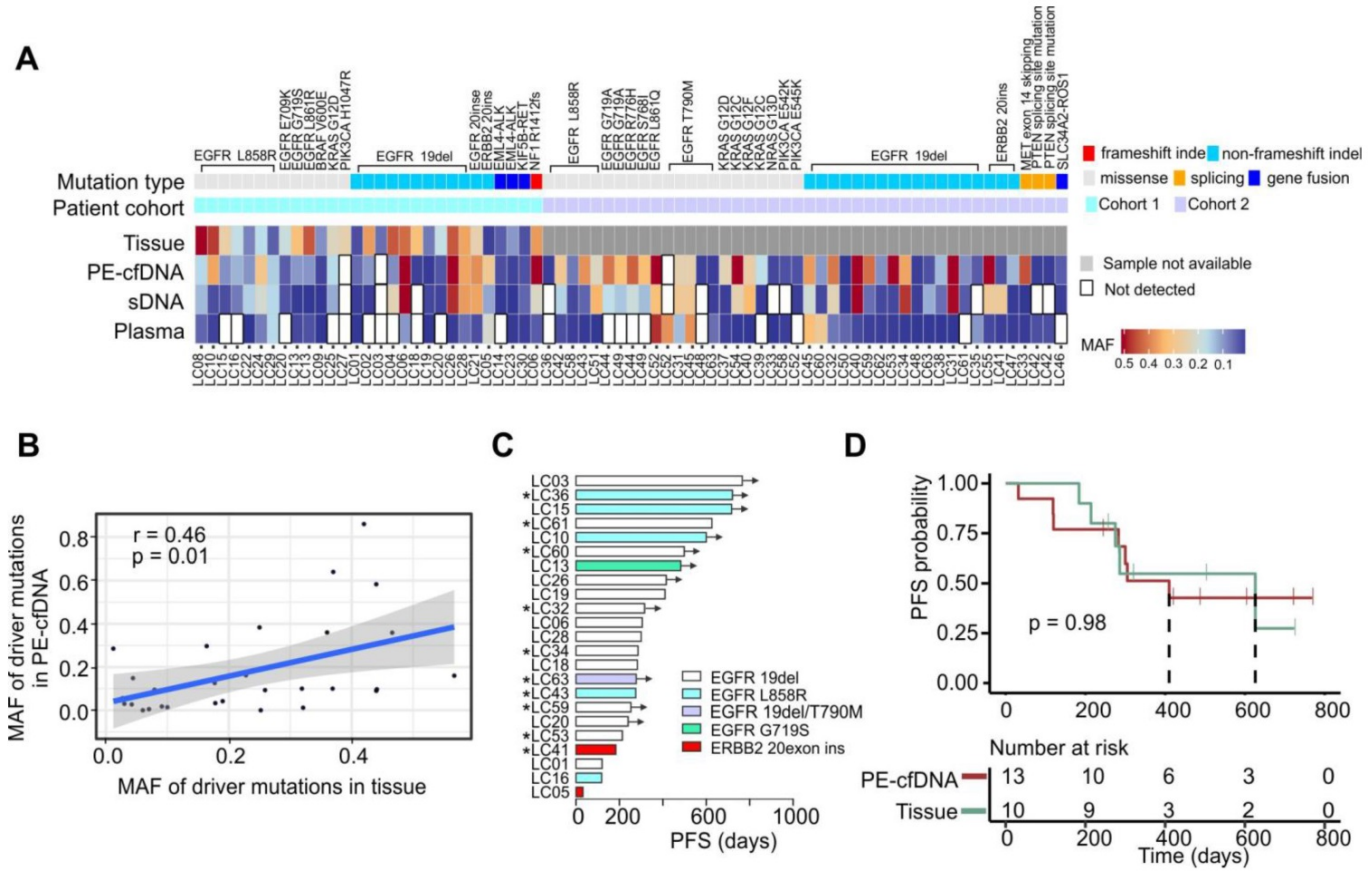
** Driver mutation detection efficiency in different samples, compared to tissue** A) The detection of driver mutations in all matched samples. MAF values are indicated by the color gradient. B) The MAF correlation of driver mutations in tissue to mutations in PE-cfDNA. The correlation analysis was performed by using Pearson correlation. C) Progression free survival (PFS) times for patients that were treated with targeted drugs following the identification of sensitive gene alterations in *EGFR* and *ERBB2*. “*” indicates that the treatment was based on the drug sensitive mutants identified in PE-cfDNA. Black arrows indicate that patients had not progressed at the time of follow-up. D) Kaplan Meier progression-free survival curve for patients that have EGFR sensitive mutants detected from PE-cfDNA or tumor tissues. P value was calculated by log rank test.

Twenty-nine driver mutations were detected from 26 tumors in cohort 1 (Figure 2A). PE-cfDNA and sDNA exhibited similar sensitivities for identifying those driver mutations (93% vs 90%), while plasma had the lowest sensitivity (63%). The MAFs of those driver mutations detected in PE-cfDNA were positively correlated to those in tissue samples (p=0.01, r=0.46, Figure 2B). However, two undetected mutations in PE-cfDNA, *EGFR* 19del (matched tissue MAF=6%) in patient LC03 and *PIK3CA* H1047R (matched tissue MAF=25%) in patient LC27, were not due to the low MAF in tissue samples. Both mutations were also missed in the sDNA and plasma cfDNA testing. In cohort 2, a total of 43 driver mutations were identified in PE-cfDNA from 31 patients, which was more than the 38 in sDNA and 24 in plasma (Figure 2A). In patients that progressed on TKI treatment, *EGFR* T790M was detected in the liquid biopsies of five post-treatment samples from patients LC31, LC45, LC48, LC52 and LC63, of which PE-cfDNA and plasma captured four and sDNA captured three (Figure 2A, Table S4).

Twenty-three patients from cohorts 1 and 2 received targeted treatments based on their genomic profiles (20 with *EGFR*-sensitizing mutations, 1 with *EGFR* secondary mutation T790M and 2 with *ERBB2* exon20 insertions), among which, 10 were treated based on the mutations detected by PE-cfDNA testing due to their tumor tissues being inaccessible (Figure 2C, marked by “*”). Patients' responses to those drugs were assessed as either a partial response or stable disease, which were aligned with their genotypes (Figure 2C). Overall, an equal progression free survival (PFS) were found between patients whose treatment decision were made based on genomic profiling from tumor tissue samples or from PE-cfDNA liquid biopsies (Figure 2D, p = 0.98).

### The mutation detection sensitivity of PE-cfDNA is independent of detectable effusion tumor cells and blood cell contamination in PE

**Figure 3:**
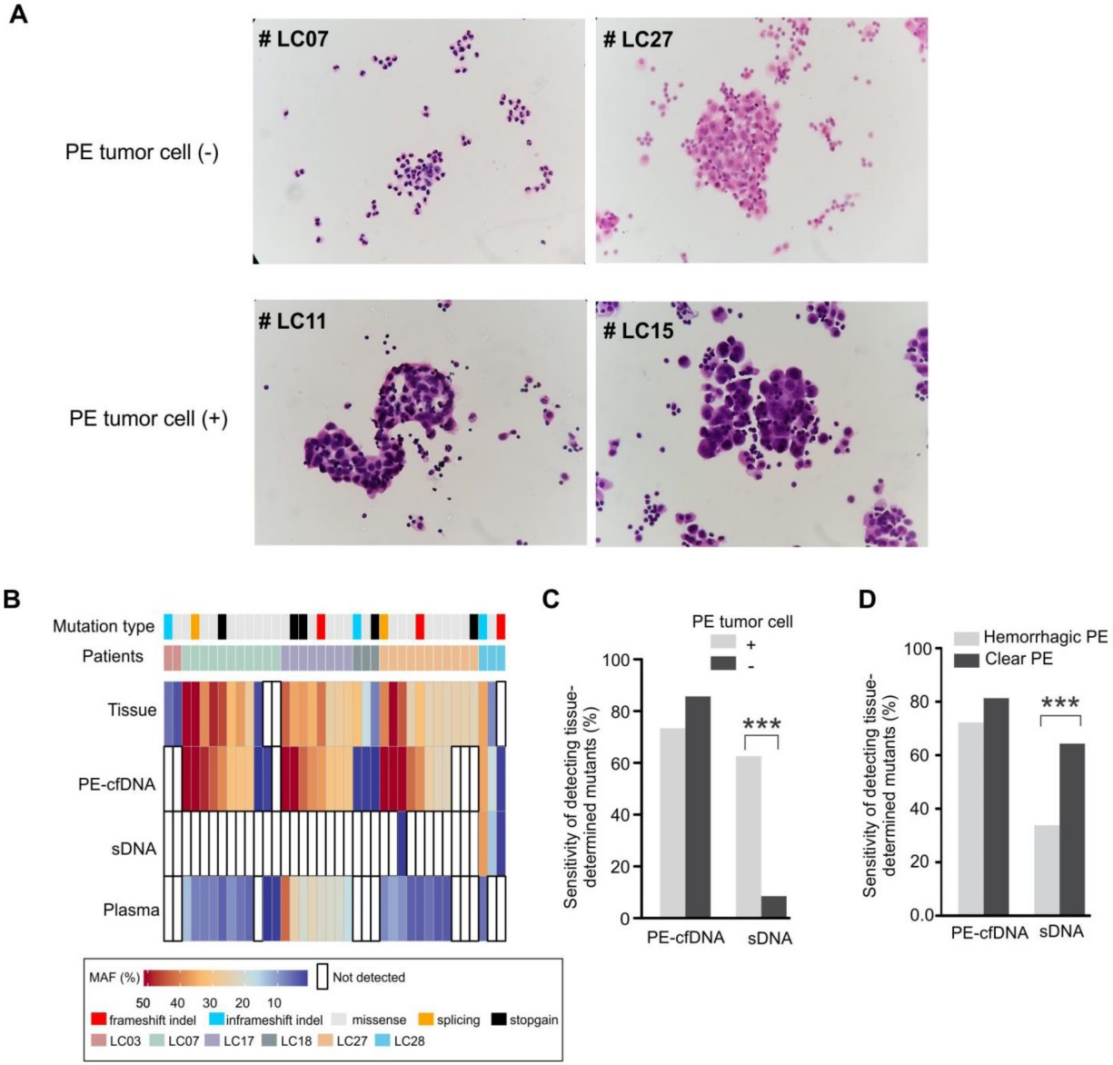
** The mutation detection of cytological negative PE and hemorrhagic PE.** A) Cytological inspection of cells in pleural effusion. For each pleural effusion sample, a haemotoxylin and eosin (H&E) stained cell smear was observed under the microscope at 400× magnification. Tumor cell(-): samples without tumor cells; tumor cell(+): samples with tumor cells. B) Mutation spectra of PE-cfDNA and sDNA from six tumor cell(-) PE samples. C) In cohort 1, the sensitivities of detecting tissue-determined mutations in PE-cfDNA and sDNA from tumor cell(+) or tumor cell(-) pleural effusion. The Chi-square test was used to compare the sensitivity between tumor cell(+) and tumor cell(-) samples. D) In cohort 1, the sensitivities of detecting tissue-determined mutations in PE-cfDNA and sDNA from hemorrhagic or clear pleural effusion. ***, p < 0.001.

The abundance of tumor cells is critical for mutation detection in sDNA. Six PE samples (patients LC03, LC07, LC17, LC18, LC27 and LC28) contained only mesothelial or inflammation-related cells, but no tumor cells (Figure 3A). For these six tumor cell(-) samples, only three mutations from two patients were detected in PE-cell sediments, accounting for 9% of the total mutations detected in their matched tumor tissues. Conversely, PE-cfDNA from those samples recovered 86% of tumor mutations from five patients (Figure 3B, 3C), suggesting that cytologically negative PE still contained sufficient PE-cfDNA for mutation profiling. Of note, three of the PE tumor cell(-) patients (LC07, LC17, and LC27) were diagnosed with small cell lung cancer, which is less likely to develop malignant PE compared to adenocarcinomas^32^, but their PE-cfDNA still contained trace amounts of tumor cfDNA. After excluding the six tumor cell(-) patients, mutation detection in PE-cfDNA remained superior to sDNA (p=0.027) (Table 2).

Hemorrhagic PE contains additional nucleated cells (lymphocytes, neutrophils and monocyte/macrophages)^33^, which reduced the sensitivity of somatic mutation detection in sDNA by diluting the tumor cells with normal cells, but not in PE-cfDNA testing (Figure 3D). PE-cfDNA demonstrated similar sensitivities of mutation detection between hemorrhagic and clear PE (72% vs. 81%), while sDNA had substantially lower sensitivities in hemorrhagic samples compared to clear samples (34% vs. 64%, p<0.001).

### A case study revealed spatial and dynamic genomic profiling of PE-cfDNA in a patient with PE from both sides of the pleural cavity

**Figure 4:**
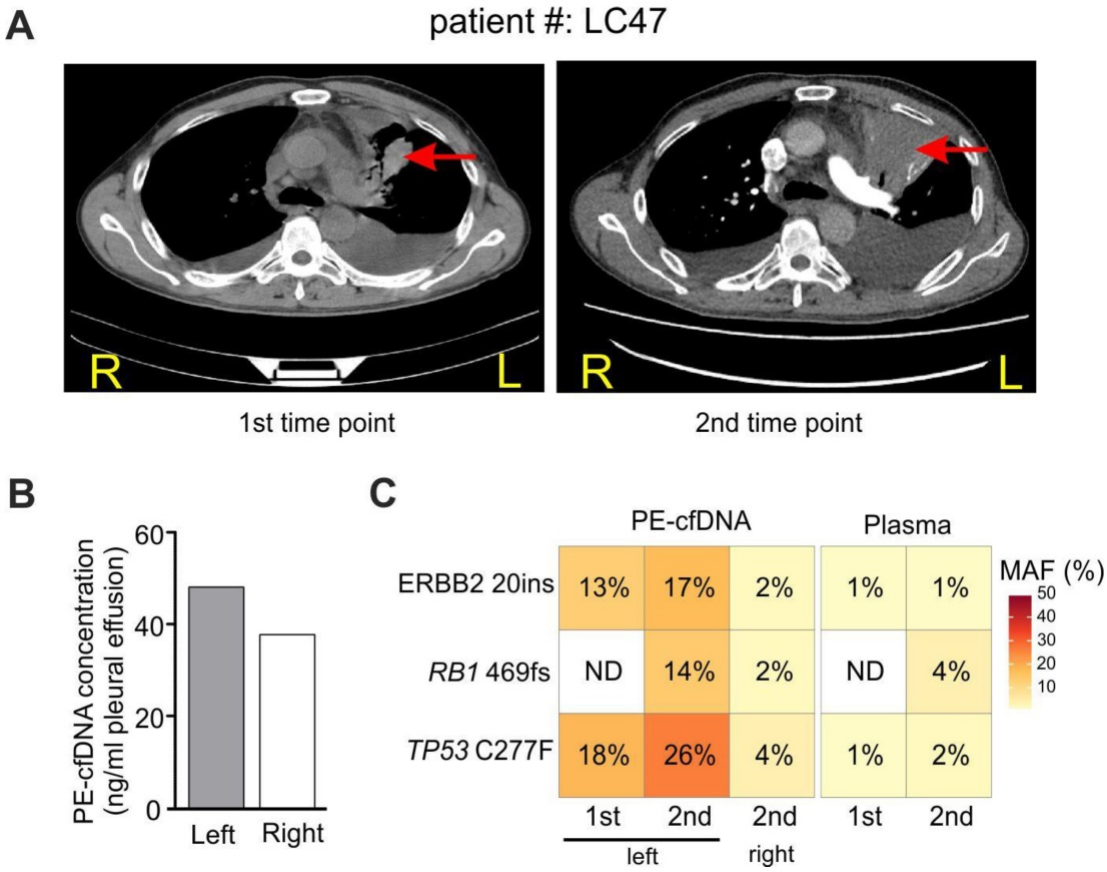
** Tumor location influences tumor content in pleural effusion.** A) CT imaging of patient #LC47 at two time points. Red arrows indicate primary tumor lesions. R, right; L, left. B) The concentration of cfDNA extracted from the pleural effusion of two separate pleural cavities. C) Samples from the right and left pleural cavities were tested independently. 1^st^ and 2^nd^, correspond to the 1^st^ and 2^nd^ time points in A).

For patient LC47 (M1a stage), the major tumor burden was in the upper lobe of the left lung, and the patient developed PE in both sides of the pleural cavity (Figure 4A). During two follow-up examinations two months apart, computed tomography (CT) imaging revealed that the tumor extended into the left chest wall. PE was collected from the left pleural cavity during the first examination, and was collected from both sides during the second examination since the two pleural cavities were isolated. Similar PE-cfDNA concentrations were obtained from the left and right pleural cavities (Figure 4B); however, the MAFs of tumor-determined mutations were higher in samples from the left side containing the tumor (Figure 4C). The left PE-cfDNA from the second examination had higher MAFs compared to samples obtained during the first examination, which was consistent with the enlarged tumor occupancy observed by CT imaging (Figure 4A). Despite the change in tumor size, the MAFs from plasma samples increased minimally except for the newly acquired *RB1* mutation, suggesting plasma was relatively insensitive to local disease progression.

## Discussion

Thoracentesis is a common operation to relieve respiratory distress in patients with large amounts of PE. As a result of the procedure, PE-cfDNA can be extracted from the fluid sample and act as a potential substitute for genetic profiling when tumor tissue biopsies are unavailable, or as an auxiliary diagnostic tool in addition to tumor tissues. PE cfDNA concentrations and MAFs were significantly higher than that of plasma cfDNA, suggesting more abundant tumor-derived DNA in PE supernatants. This indicates that analysis of PE-cfDNA is more representative of tumor heterogeneity and provides more reliable results^34^, ^35^.

Different mutation types, including SNVs, indels, fusions, and splicing variants were detected in PE-cfDNA. When comparing TMB levels, an independent biomarker of response to immunotherapy^36^, between PE-cfDNA, sDNA and plasma, it was shown that TMB in PE-cfDNA was more similar to tissue TMBs than to sDNA and plasma. Thus PE-cfDNA also provides more accuracy for identifying immunotherapy-responsive patients.

Hemorrhagic PE is frequently observed in advanced lung cancer patients. The presence of blood cells in PE can interfere with the detection of tumor mutants^33^, but unlike PE cell sediments, PE-cfDNA was less affected by the presence of non-tumor cell components. This was demonstrated through high levels of mutational spectrum congruency between PE-cfDNA and tumor tissues. Another advantage of PE-cfDNA is the ability to detect tumor mutants from cytologically negative PE. In this study, supernatant cfDNA from six cytologically negative PE samples (3 from SCLC) had higher pick-up rates (72%) of tissue-determined mutations than sDNA (34%). These findings suggest that PE-cfDNA analysis not only can reveal genetic mutations in NSCLC, but also potentially extend to SCLC and other atypical lung cancers that have fewer tumor cells in PE.

We also observed that for stage M1a patients with restricted tumor metastasis, the mutation spectrum of PE-cfDNA was concordant with that of tumor tissues, and almost equal to the sum of sDNA and plasma. This data suggest that PE-cfDNA is the most accurate representation of tumor mutations in stage M1a when tumor tissues are unavailable. In M1b/M1c stages, PE-cfDNA and sDNA samples disclosed unique and novel mutations that were absent in the matched tumor tissues, suggesting that PE-derived samples, especially PE-cfDNA, are representative of the tumor heterogeneity in advanced disease. Although the mechanism of PE formation has not been fully elucidated, it is hypothesized to be of plasma-origin due to the increased pleural vascular permeability in cancer^37^, ^38^. When possible, genetic testing of PE-cfDNA and plasma cfDNA can be combined to more accurately capture the inter-tumor heterogeneity in M1b/M1c stages.

In our case study, comparisons of PE-cfDNA from both sides of the pleural cavity revealed that the fluid from the side with pleural invasion has higher MAFs despite similar cfDNA concentrations on both sides. Thus, in clinical practice, PE collected from the pleural cavity with tumor invasion will increase the reliability and efficacy of detecting mutations.

In advanced lung cancers, cfDNA from PE supernatants proves to be more informative for tumor genomic profiling than sDNA from PE cell sediments and plasma cfDNA. Thus, PE-cfDNA could be an alternative to tumor tissues if it is shown to be a better diagnostic tool following additional validations in much larger study cohorts. Genetic profiling of such readily accessible liquid biopsy samples is particularly useful when tumor tissues are unavailable. This is not only the case for the initial diagnostic detection of sensitive mutations, but also for the identification of secondary acquired mutations in drug-resistance tumors. In addition to lung cancer, PE is also frequently observed in advanced breast cancer^39^, gastrointestinal cancer, and ovarian cancer^40^. Further investigation through comparative studies may also reveal that PE-cfDNA demonstrate similar diagnostic potential in those cancer types.

## Conclusions

PE-cfDNA can be a better candidate for genomic profiling when tumor tissues are not available than PE sDNA and plasma cfDNA.

## Supplementary Material

Supplementary figures and tables.

## Figures and Tables

**Table 1:** Sensitivities of detecting tissue-determined CNV in different sample types in Cohort 1

Tissue CNV detection			PE-cfDNA			Plasma		
			+	-	*p-value*	+	-	*p-value*
All patients in Cohort 1 (n = 30)	sDNA	+	7	2	<0.01	2	7	0.07
		-	13	42		1	54	
	Plasma	+	2	1	<0.0001			
		-	18	43				

**Table 2:** Sensitivities of detecting tissue-determined mutations in different sample types in Cohort 1

			PE-cfDNA			Plasma		
			+	-	*p-value*	+	-	*p-value*
All patients in Cohort 1 (n = 30)	sDNA	+	59	1	<0.0001	36	24	0.24
		-	36	28		34	30	
	Plasma	+	64	6	<0.0001			
		-	31	23				
M1a stage (n = 18)	sDNA	+	31	0	<0.0001	21	15	0.03
		-	35	17		32	24	
	Plasma	+	45	5	<0.0001			
		-	35	17				
M1b & M1c stage (n = 12)	sDNA	+	28	1	1	19	10	0.30
		-	1	11		5	7	
	Plasma	+	19	5	0.30			
		-	10	7				
PE tumor cell(+)* patients (n = 24)	sDNA	+	56	1	0.027	34	23	0.059
		-	9	23		11	21	
	Plasma	+	39	6	<0.001			
		-	26	18				

“+” detected; “-” not detected. * PE tumor cell(+): Tumor cells were identified in pleural effusion sample. The McNemar test was used to compare results between different samples.

## Abbreviations

PE: pleural effusion
cfDNA: cell free DNA
sDNA: sediment DNA
NGS: next-generation sequencing
TMB: tumor mutational burden
NSCLC: non-small cell lung cancer
FFPE: formalin-fixed, paraffin-embedded
GATK: Genome Analysis Toolkit
MAF: mutant allele frequency
CNV: copy number variations
CIN: chromosomal instability
ARMS: amplification-refractory mutation system
