# Supplementary material for: Tumor-derived DNA from pleural effusion supernatant as a promising alternative to tumor tissue in genomic profiling of advanced lung cancer
Source: Theranostics. 2019 Jul 28;9(19):5532–41. doi: 10.7150/thno.34070 (PMC6735385; doi:10.7150/thno.34070)
Supplement: Supplementary file 1 — Supplementary figures and tables. [file thnov09p5532s1.pdf]

Tumor-derived DNA from pleural effusion supernatant as a promising alternative to tumor tissue in genomic profiling of advanced lung cancer

Lin Tong, Ning Ding, Xiaoling Tong, Jiamin Li, Yong Zhang, Xiaodan Wang, Xiaobo Xu, Maosong Ye, Chun Li, Xue Wu, Hairong Bao, Xin Zhang, Qunying Hong, Yuanlin Song， Yang W. Shao, Chunxue Bai, Jian Zhou, Jie Hu

Online Data Supplement

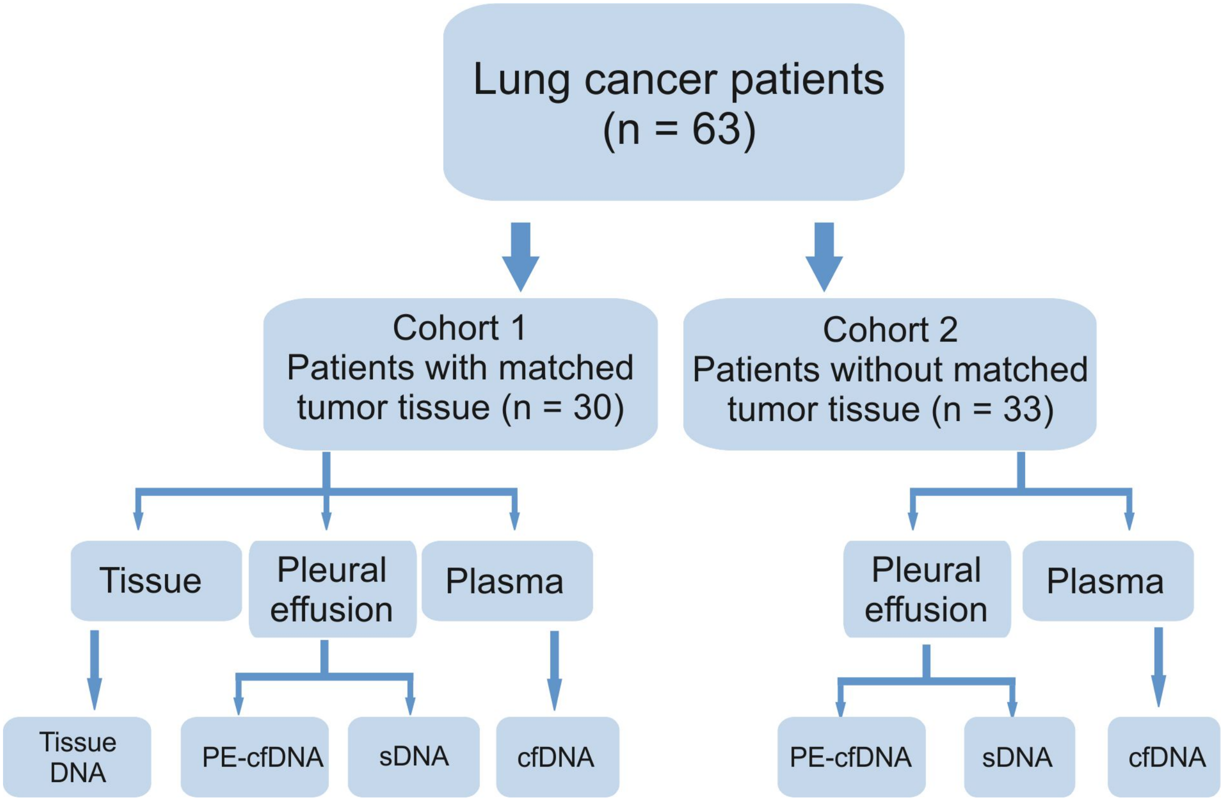

Figure S1. Study design for patient enrollment and sample collection. Of 63 eligible patients, 30 patients with matched tissue samples available were grouped as Cohort 1, while the remaining 33 patients were grouped as Cohort 2. DNA was extracted from different sample types for targeted NGS analysis.

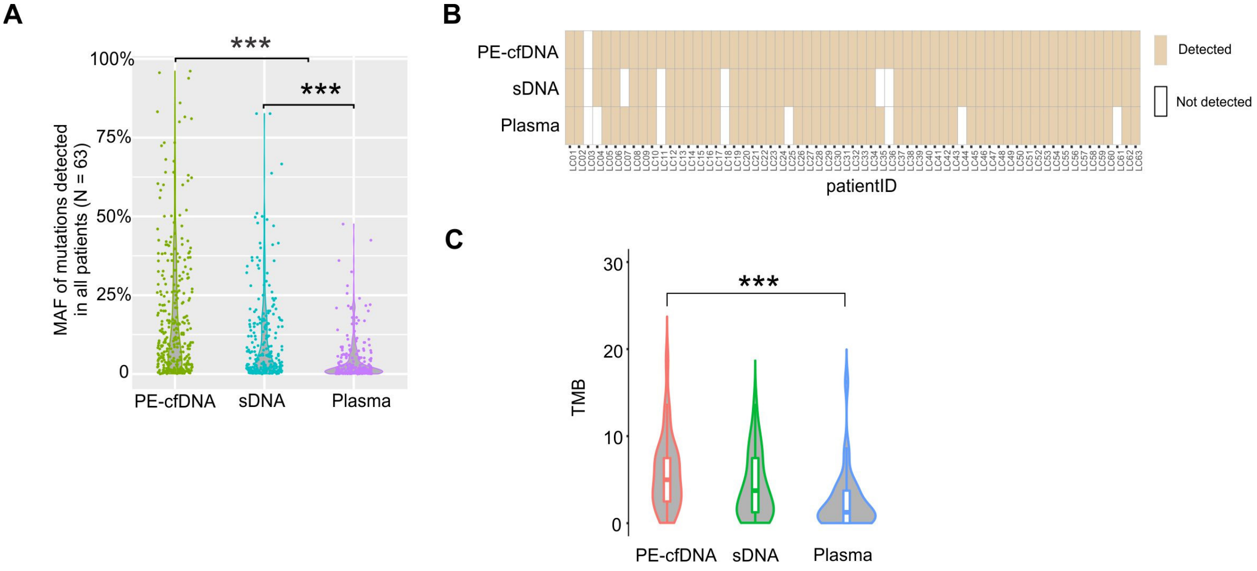

Figure S2. The gene variation detection in entire study cohort and TMB in cohort 2. A) MAFs of all detected mutations in different sample types from 63 patients. B) The variation detection status in all patients. Patients with at least one gene variation detected were marked as ‘Detected’ in the plot. C) TMB in different sample types from Cohort 2. For statistic test in A) and C), the One-way ANOVA on ranks test was used to compare all groups and the Dunn’s test was used for post-hoc analyses to compare matched groups. ***, p < 0.001.

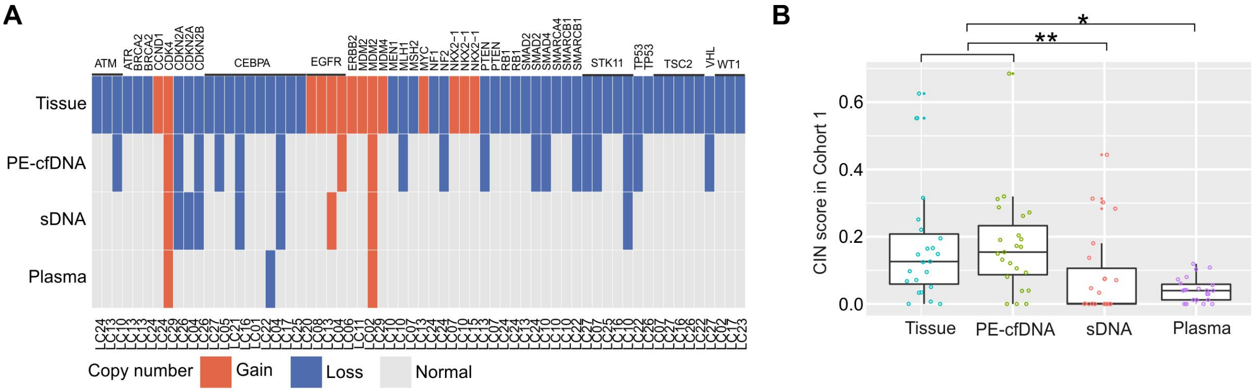

Figure S3. CNV and the proportion of unstable chromosome segments detection in different sample types from patients in Cohort 1. A) In the 30 patients of Cohort 1, CNVs from each sample type were grouped and plotted together. Each column represents the CNV status of a specific gene in a single patient. B) CIN scores were calculated as the proportion of unstable chromosome segments in different sample types in Cohort 2. Each dot represents one patient. PE-cfDNA showed similar CIN scores to tissue samples, while both were significantly higher than those of sDNA and plasma cfDNA samples (Kruskal–Wallis one-way analysis of variance and Dunn's multiple comparison tests were used for statistical analysis). *, $p < 0.05$; **, $p < 0.01$.

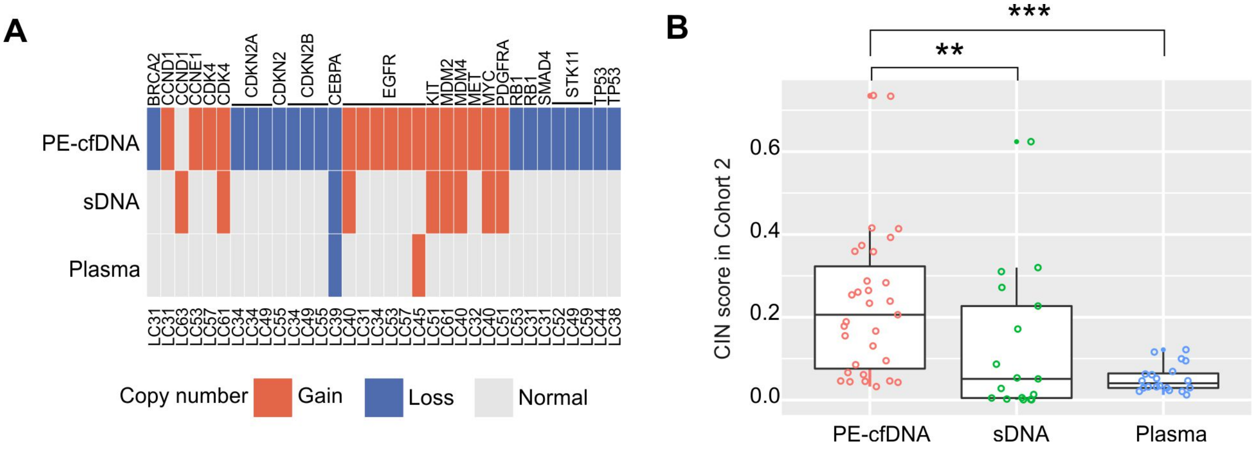

Figure S4. CNV and CIN detection in different sample types from patients in Cohort 2. A) CNVs from each sample type in Cohort 2 were plotted for comparison. Each column represents the CNV status of a specific gene in a single patient. B) CIN score as calculated by the proportion of unstable chromosome segments in different sample types. Each dot represents one patient. PE-cfDNA showed significantly higher CIN scores than sDNA and plasma cfDNA samples (Kruskal–Wallis one-way analysis of variance and Dunn's multiple comparison tests were used for statistical analysis). **, $p < 0.01$, ***, $p < 0.001$.

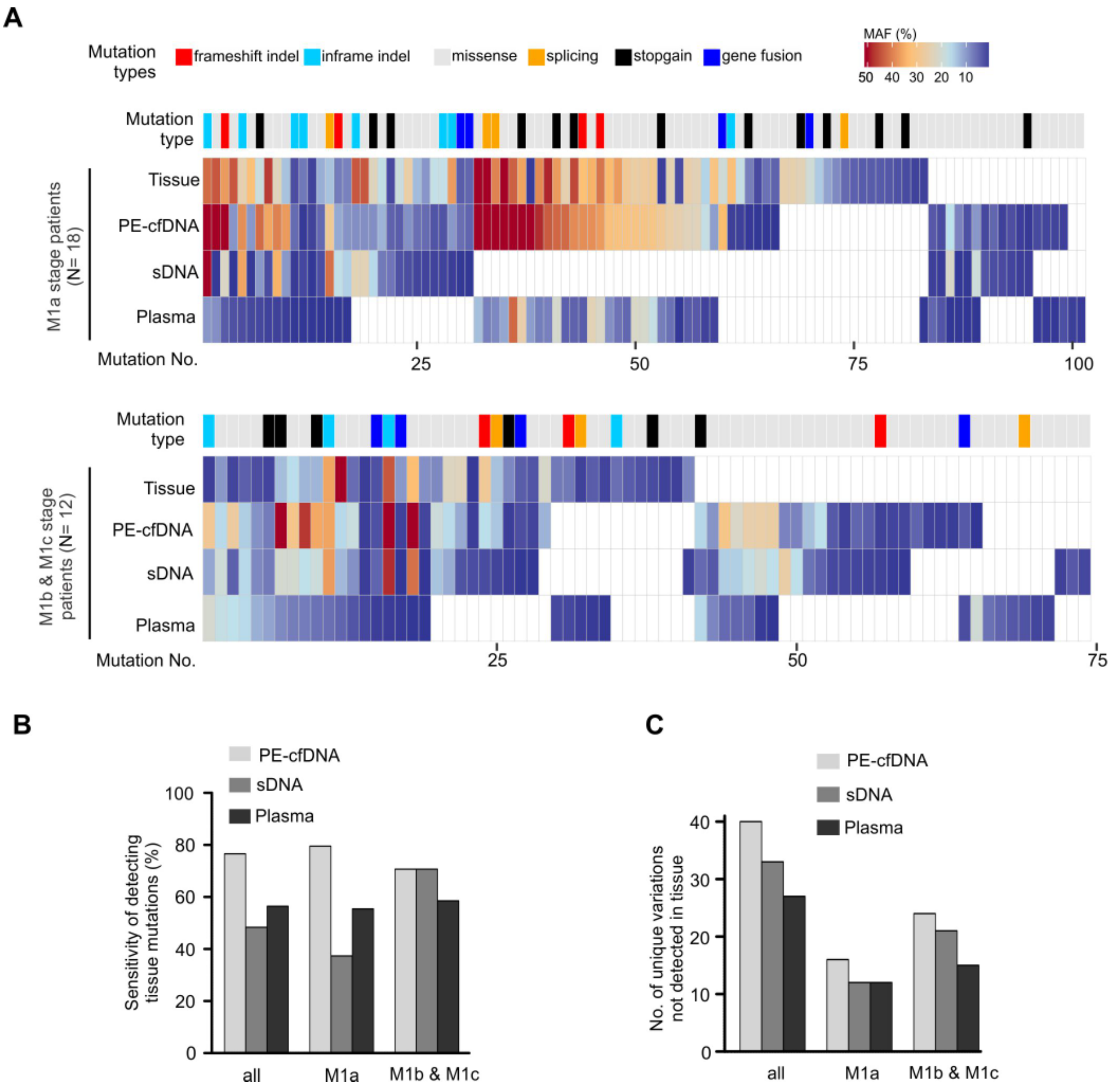

Figure S5. Unique and shared mutations across different sample types. A) Patients in Cohort 1 were divided into M1a and M1b&M1c according to their disease stages. Mutations in different sample types were grouped and plotted with their MAF values marked as gradient colors. Each column represents one mutation and the mutation type is marked on the top of the panel. B) Sensitivities of detecting tissue-determined mutations in different sample types in Cohort 1 patients. C) Number of unique mutations (mutations that were not detected in tumor tissues) detected in different sample types. patients.

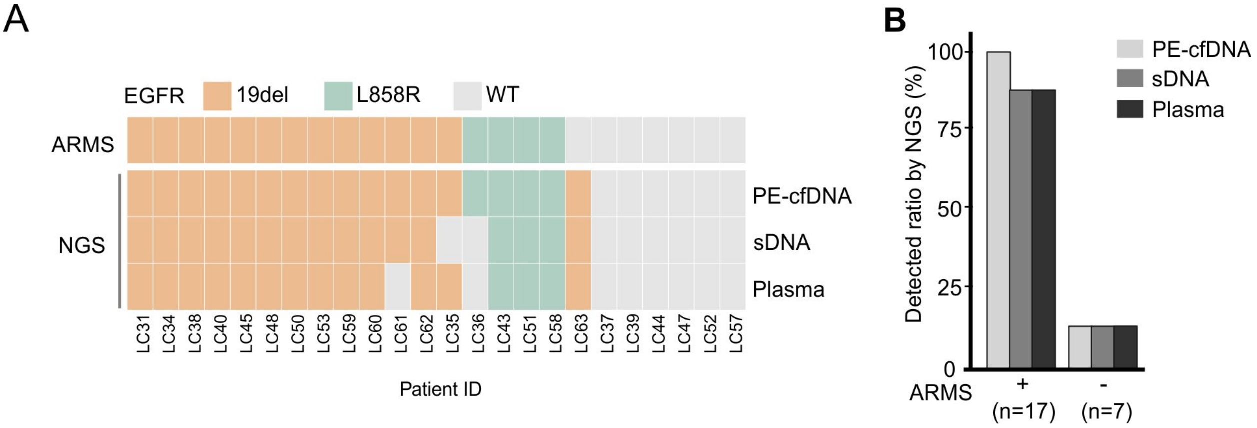

Figure S6. Comparing the detection efficiency of *EGFR* mutations by NGS and ARMS PCR methods. A) *EGFR* mutation detected by ARMS and NGS in different samples. B) The overall detection rate of NGS. ARMS(+) means *EGFR* 19del or L858R were detected, while ARMS(-) means wild-type *EGFR*. NGS of the two groups in different sample types shows the ability to recover extra *EGFR* mutations from ARMS(-).

Table S1. Genes covered by targeted NGS panel

ABCB1(MDR1)	CDKN1A	ERC1	HSD3B1	MTOR	PRSS1	STRN
ABCC2(MRP2)	CDKN1B	ERCC1	IDH1	MUTYH	PTCH1	STT3A
ADH1B	CDKN1C	ERCC2	IDH2	MYC	PTEN	SUFU
AFF1	CDKN2A	ERCC3	IGF1R	MYCL	PTK2	TACC1
AFF4	CDKN2B	ERCC4	IGF2	MYCN	PTPN11	TACC3
AIP	CDKN2C	ERCC5	IKBKE	MYD88	PTPRD	TEK
AKT1	CEBPA	ERG	IKZF1	NAT1	QKI	TEKT4
AKT2	CEP57	ESR1	IKZF3	NBN	RAC1	TERC
AKT3	CHD4	ETV1	IL7R	NCOA4	RAD50	TERT
ALDH2	CHEK1	ETV4	INPP4B	NF1	RAD51	TET2
ALK	CHEK2	ETV6	INPP5D	NF2	RAD51C	TFG
AMER1	CLIP1	EWSR1	IRF2	NFKBIA	RAD51D	TGFBR2
APC	CLTC	EXT1	JAK1	NKX2-1	RAF1	THADA
AR	COL1A1	EXT2	JAK2	NOTCH1	RARA	TMEM127
ARAF	CREB1	EZH2	JAK3	NOTCH2	RB1	TMPRSS2
ARID1A	CREBBP	EZR	JUN	NPM1	RECQL4	TNFAIP3
ARID2	CRKL	FANCA	KDM5A	NQO1	RET	TNFRSF11A
ARID5B	CSF1R	FANCC	KDM6A	NR4A3	RHOA	TNFRSF14
ASXL1	CTCF	FANCD2	KDR(VEGFR2)	NRAS	RICTOR	TNFRSF19
ATF1	CTLA4	FANCE	KIF5B	NSD1	RNF146	TNFSF11
ATIC	CTNNB1	FANCF	KIT	NTRK1	RNF43	TOP1
ATM	CXCR4	FANCG	KITLG	PAK3	ROS1	TOP2A
ATR	CYLD	FANCL	KLC1	PALB2	RPTOR	TP53
ATRX	CYP19A1	FAT1	KLLN	PALLD	RRM1	TPM3
AURKA	CYP2A6	FBX1	KMT2A	PARK2	RTEL1	TPM4
AURKB	CYP2B6*6	FBXW7	KMT2B	PARP1	RUNX1	TPMT*2
AXIN2	CYP2C19*2	FEV	KRAS	PARP2	SBDS	TPMT*3
AXL	CYP2C9*3	FGF19	KTN1	PAX5	SDC4	TPMT*4
BAIAP2L1	CYP2D6*3	FGFR1	LHCGR	PBRM1	SDHA	TPMT*5
BAK1	CYP2D6*4	FGFR2	LMO1	PCDH11Y	SDHAF2	TPMT*6
BAP1	CYP2D6*5	FGFR3	LRIG3	PDCD1 (PD1)	SDHB	TPMT*7
BARD1	CYP2D6*6	FGFR4	LYN	PDCD1LG2(PD-L2)	SDHC	TPMT*10
BCL2	CYP2D6*7	FH	LZTR1	PDE11A	SDHD	TRIM24
BCL2L11(BIM)	CYP2D6*11	FLCN	MAP2K1(MEK1)	PDGFRA	SEPT9	TRIM27
BIRC3	CYP2D6*12	FLI1	MAP2K2(MEK2)	PDGFRB	SERP2	TRIM33
BLM	CYP2D6*14	FLT1(VEGFR1)	MAP2K4	PDK1	SETBP1	TSC1

BMPR1A	CYP3A4*4	FLT3	MAP3K1	PGR	SETD2	TSC2
BRAF	CYP3A5*1	FLT4	MAP4K3	PHOX2B	SF3B1	TSHR
BRCA1	CYP3A5*3	GATA1	MAX	PIK3C3	SGK1	TTF1
BRCA2	DAXX	GATA2	MCL1	PIK3CA	SH2D1A	TUBB3
BRD4	DCTN1	GATA3	MDM2	PIK3R1	SHOX	TYMS
BRIP1	DDIT3	GATA4	MDM4	PIK3R2	SLC34A2	UGT1A1
BTG2	DDR2	GATA6	MED12	PKD1	SLC7A8	VEGFA
BTK	DENND1A	GNA11	MEF2B	PKD2	SLX4	VHL
BUB1B	DHFR	GNAQ	MEN1	PKHD1	SMAD2	WAS
c11orf30	DICER1	GNAS	MET	PLAG1	SMAD3	WISP3
CBL	DNMT3A	GOLGA5	MGMT	PLK1	SMAD4	WRN
CBLB	DPYD	GOPC	MITF	PMS1	SMAD7	WT1
CCND1	DUSP2	GRIN2A	MLH1	PMS2	SMARCA4	XPA
CCNE1	EGFR	GRM3	MLH3	POLD1	SMARCB1	XPC
CD274(PD-L1)	EML4	GSTM1	MLLT1	POLE	SMO	XRCC1
CD74	EP300	GSTP1	MLLT10	POLH	SOX2	YAP1
CDA	EPAS1	GSTT1	MLLT3	POT1	SPOP	ZNF2
CDC73	EPCAM	HDAC2	MLLT4	POU5F1	SPRY4	ZNF217
CDH1	EPHA2	HGF	MPL	PPP2R1A	SRC	ZNF444
CDK10	EPHA3	HIP1	MRE11A	PRDM1	SRY	ZNF703
CDK12	EPS15	HLA-A	MSH2	PRF1	STAG2	
CDK4	ERBB2(HER2)	HNF1A	MSH3	PRKACA	STAT3	
CDK6	ERBB3	HNF1B	MSH6	PRKAR1A	STK11	
CDK8	ERBB4	HRAS	MTHFR	PRKCI	STMN1	

Table S2. Demographic and clinical characteristics of patients

Characteristics	Patient No. = 63	%
**Age (years)**		
Median	64	-
Range	34-96	-
**Gender**		
Female	28	44.4
Male	35	55.6
**Histological classification**		
Adenocarcinoma	58	92.1
Adeno-squamous carcinoma	2	3.2
Small cell lung cancer	3	4.8
**Clinical stage**		
M1a	35	55.6
M1b	7	11.1
M1c	21	33.3
**Smoking history**		
Never	43	68.3
Current	13	20.6
Former	7	11.1
**Systemic treatment**		
Untreated	42	66.7
First line	11	17.4
Second line and beyond	10	15.9
**Hemorrhagic pleural effusion**		
Yes	31	49.2
No	32	50.8
**Effusion tumor cell**		
Positive	57	90.5
Negative	6	9.5

Table S3. Clinical information of each patient

Patient ID	Sex	Age	Stage	M stage	Histology	Smoking history	Effusion tumor cell in PE	Hemorrhagic PE	Tissue sampling site	Treatment received
LC01	male	67	cT4N0M1a	M1a	adenocarcinoma	never	yes	no	pleura	No
LC02	female	71	cT3N3M1a	M1a	adenocarcinoma	never	yes	yes	lung	First line
LC03	male	70	cT4N2M1c	M1c	adenocarcinoma	never	no	no	lung	Second line or more
LC04	female	61	rT2N0M1a	M1a	adenocarcinoma	never	yes	no	lung	No
LC05	male	64	cT2aN3M1c	M1c	adenocarcinoma	never	yes	no	lung	No
LC06	female	62	cT4N0M1a	M1a	adenocarcinoma	never	yes	no	pleura	No
LC07	male	77	cT4N1M1a	M1a	small cell	current	no	yes	lung	No
LC08	male	60	cTxN2M1c	M1c	adenocarcinoma	never	yes	no	pleura	Second line or more
LC09	male	72	cT2aN3M1b	M1b	adenocarcinoma	former	yes	yes	lung	No
LC10	female	55	cT4N2M1a	M1a	adenocarcinoma	never	yes	yes	lung	No
LC11	female	49	cT1cN3M1a	M1a	adenocarcinoma	never	yes	yes	pleura	Second line or more
LC12	male	53	cT1cN2M1a	M1a	adenocarcinoma	current	yes	no	lymph node	No
LC13	female	69	rT2N0M1a	M1a	adenocarcinoma	never	yes	yes	lung	No
LC14	female	45	cT3N3M1b	M1b	adenocarcinoma	current	yes	no	lung	First line
LC15	female	79	cT3N3M1a	M1a	adenocarcinoma	never	yes	no	lymph node	No
LC16	male	78	cT2bN0M1a	M1a	adenocarcinoma	never	yes	no	lung	No
LC17	male	44	cT2bN2M1a	M1a	small cell	former	no	no	lung	No
LC18	female	66	cT4N1M1a	M1a	adenocarcinoma	never	no	yes	lymph node	No
LC19	female	85	cT2bN3M1a	M1a	adenocarcinoma	never	yes	yes	lymph node	No
LC20	female	64	cT2bN3M1a	M1a	adenocarcinoma	never	yes	yes	lymph node	No
LC21	male	68	rT2N1M1a	M1a	adenocarcinoma	former	yes	no	lung	No

LC22	female	66	cT2bN2M1a	M1a	adenocarcinoma	never	yes	yes	lung	Second line or more
LC23	male	34	yT4N2M1c	M1c	adenocarcinoma	never	yes	no	lung	Second line or more
LC24	male	57	cT2bN2M1c	M1c	Adeno-squamous	current	yes	no	lung	First line
LC25	female	64	cT2aN2M1c	M1c	adenocarcinoma	never	yes	no	lung	No
LC26	female	65	rT1bN3M1c	M1c	adenocarcinoma	never	yes	yes	lung	No
LC27	male	56	cT4N3M1a	M1a	small cell	current	no	yes	lung	No
LC28	male	49	cT2N2M1c	M1c	Adeno-squamous	never	no	yes	lung	No
LC29	male	64	cT1cN2M1c	M1c	adenocarcinoma	former	yes	no	lung	Second line or more
LC30	male	58	cT2bN2M1b	M1b	adenocarcinoma	never	yes	no	lung	No
LC31	female	44	cT4N0M1a	M1a	adenocarcinoma	never	yes	no	NA	First line
LC32	female	65	cT2aN0M1c	M1c	adenocarcinoma	never	yes	no	NA	No
LC33	male	62	cT4N2M1a	M1a	adenocarcinoma	current	yes	yes	NA	No
LC34	male	46	cT3N3M1c	M1c	adenocarcinoma	current	yes	yes	NA	No
LC35	female	69	cT4N3M1c	M1c	adenocarcinoma	never	yes	yes	NA	No
LC36	male	72	cT1N2M1c	M1c	adenocarcinoma	former	yes	yes	NA	First line
LC37	female	66	cT2bN0M1b	M1b	adenocarcinoma	never	yes	no	NA	No
LC38	male	68	cT2aN3M1c	M1c	adenocarcinoma	current	yes	yes	NA	No
LC39	male	54	cT1bN2M1b	M1b	adenocarcinoma	current	yes	no	NA	No
LC40	male	76	cT1bN0M1b	M1b	adenocarcinoma	never	yes	yes	NA	No
LC41	male	56	cT2bN3M1a	M1a	adenocarcinoma	current	yes	no	NA	No
LC42	male	79	cT1cN0M1a	M1a	adenocarcinoma	never	yes	yes	NA	First line
LC43	female	59	cT1bN2M1a	M1a	adenocarcinoma	never	yes	no	NA	No
LC44	female	63	cTxN0M1a	M1a	adenocarcinoma	current	yes	yes	NA	No
LC45	male	78	yT1cN0M1c	M1c	adenocarcinoma	never	yes	no	NA	Second line or more
LC46	female	96	cT1bN2M1a	M1a	adenocarcinoma	never	yes	yes	NA	No
LC47	male	49	cT2bN2M1a	M1a	adenocarcinoma	former	yes	yes	NA	No

LC48	male	58	cTxN3M1a	M1a	adenocarcinoma	current	yes	no	NA	Second line or more
LC49	female	80	rT4N3M1a	M1a	adenocarcinoma	never	yes	yes	NA	First line
LC50	female	61	cT4N3M1a	M1a	adenocarcinoma	never	yes	yes	NA	Second line or more
LC51	male	59	cT3N3M1a	M1a	adenocarcinoma	never	yes	yes	NA	First line
LC52	female	73	cT4N2M1c	M1c	adenocarcinoma	never	yes	yes	NA	Second line or more
LC53	female	60	cT3N0M1a	M1a	adenocarcinoma	never	yes	yes	NA	First line
LC54	male	65	cT3N3M1a	M1a	adenocarcinoma	former	yes	no	NA	No
LC55	male	75	cT2aN0M1c	M1c	adenocarcinoma	never	yes	no	NA	First line
LC56	male	76	cT2aN0M1c	M1c	adenocarcinoma	current	yes	no	NA	No
LC57	male	53	cT1bN2M1b	M1b	adenocarcinoma	never	yes	yes	NA	No
LC58	male	69	cT2N3M1c	M1c	adenocarcinoma	never	yes	no	NA	No
LC59	female	55	cT1bN2M1a	M1a	adenocarcinoma	never	yes	no	NA	No
LC60	male	62	cT2aN3M1c	M1c	adenocarcinoma	never	yes	no	NA	No
LC61	female	50	cT3N0N0M1a	M1a	adenocarcinoma	never	yes	yes	NA	No
LC62	male	49	cTxN2M1c	M1c	adenocarcinoma	never	yes	yes	NA	No
LC63	female	77	rT3N3M1a	M1a	adenocarcinoma	never	yes	no	NA	First line

Table S4. Somatic mutation identified in all the sample types in each patient

patient ID	gene	amino acid change	nucleotide change	MAF			
				plasma	PE-cfDNA	sDNA	tissue
LC01	*CTNNB1*	p.D32H	c.G94C	0.20%	1.57%	0.30%	1.50%
	EGFR	p.745_750del	c.2235_2249delGGAATTAAGAGAAGC	0.30%	5.60%	0.80%	2.70%
LC02	*CTNNB1*	p.G34R	c.G100C	0.10%	3.00%	0.40%	-
	CTNNB1	p.S37Y	c.C110A	-	1.00%	1.00%	-
	EGFR	p.746_750del	c.2236_2250delGAATTAAGAGAAGCA	-	10.00%	1.00%	36.90%
	TMPRSS2	BMP7&MIR4325-TMPRSS2	BMP7&MIR4325-TMPRSS2	-	6.00%	0.40%	4.20%
	TMPRSS2	TMPRSS2-PHACTR3	TMPRSS2-PHACTR3	-	5.00%	0.20%	5.10%
LC03	*APC*	p.T2248S	c.A6742T	-	-	-	3.00%
	EGFR	p.746_750del	c.2236_2250del	-	-	-	6.00%
LC04	*EGFR*	p.ELREATS746delinsA	c.2237_2254delAATTAAGAGAAGCAACAT	-	9.00%	24.00%	44.00%
	EGFR	p.S752F	c.C2255T	-	9.00%	24.00%	44.00%
	RUNX1	p.S141X	c.C422A	-	9.00%	16.00%	23.00%
LC05	*AKT1*	p.S137Y	c.C410A	-	5.09%	1.78%	-
	ATM	NA	c.G3994-1T	3.21%	-	-	-

	ERBB2	p.G776delinsVC	c.2326_2327insTCT	20.76%	28.51%	11.42%	1.20%
	ERBB3	p.D229H	c.G685C	4.90%	-	-	-
	INPP4B	p.L809V	c.C2425G	8.21%	9.59%	3.76%	-
	JAK2	p.E90X	c.G268T	15.87%	15.77%	5.43%	-
	KDM5A	p.L1062V	c.C3184G	-	4.26%	1.37%	-
	KMT2B	p.E1214K	c.G3640A	1.59%	-	-	-
	KMT2B	p.V1828G	c.T5483G	0.70%	-	-	4.20%
	LHCGR	p.E323K	c.G967A	-	3.85%	2.05%	-
	MED12	p.L1300V	c.C3898G	-	16.85%	4.62%	-
	MPL	p.R90Q	c.G269A	-	1.64%	0.65%	-
	MYCL	NA	MYCL-ELAVL4&DMRTA2	2.50%	3.70%	-	-
	MYD88	p.L265P	c.T794C	-	-	1.31%	-
	PKHD1	p.Q716E	c.C2146G	-	1.80%	0.41%	-
	POLD1	p.H202Y	c.C604T	3.59%	-	-	-
	PTPN11	p.E123Q	c.G367C	-	13.75%	6.30%	-
	TP53	p.R280T	c.G839C	16.87%	26.98%	4.55%	1.80%
LC06	*EGFR*	p.745_750del	c.2235_2249delGGAATTAAGAGAAGC	10.00%	86.00%	51.00%	42.00%
	NF1	p.R1412fs	c.4236_4239delATTT	4.00%	64.00%	22.00%	37.00%
	RB1	p.R787X	c.C2359T	2.00%	42.00%	10.00%	19.00%
	TP53	p.E358Q	c.G1072C	1.00%	37.00%	10.00%	14.00%
	TP53	p.Q167X	c.C499T	-	-	-	11.00%
LC07	*BRCA1*	p.Q1240X	c.C3718T	4.76%	38.81%	-	45.40%
	CTCF	p.T374A	c.A1120G	1.14%	-	-	-
	EPHA3	p.D879E	c.C2637A	6.42%	31.27%	-	37.00%

	FLT1	p.R508L	c.G1523T	7.71%	43.51%	-	48.40%
	FLT3	p.E293Q	c.G877C	-	0.22%	-	2.20%
	GRIN2A	p.G591V	c.G1772T	5.23%	30.92%	-	28.30%
	JAK1	p.Y200C	c.A599G	7.46%	48.06%	-	38.80%
	PDE11A	p.N713I	c.A2138T	0.37%	1.67%	-	-
	RB1	NA	c.A1216-2G	7.70%	83.21%	-	80.30%
	SMO	p.P747L	c.C2240T	9.14%	31.71%	-	33.60%
	TP53	p.C242Y	c.G725A	13.59%	93.83%	-	88.40%
LC08	*BLM*	p.V1103I	c.G3307A	-	0.94%	-	-
	EGFR	p.L858R	c.T2573G	4.95%	16.00%	13.07%	56.70%
	FAT1	p.R4208F	c.CGG12622_12624TTT	5.11%	21.79%	14.96%	-
	MYCL	p.V21A	c.T62C	5.53%	30.16%	15.03%	-
	SDHB	p.M247V	c.A739G	5.10%	25.59%	18.02%	-
	TP53	p.K351X	c.A1051T	6.28%	35.68%	19.15%	12.80%
LC09	*BRAF*	p.V600E	c.T1799A	0.10%	1.76%	0.23%	9.10%
	CREBBP	p.S2377L	c.C7130T	0.28%	-	-	1.60%
	MLH3	p.R1115X	c.C3343T	11.00%	7.25%	7.09%	1.60%
	TP53	p.Y234C	c.A701G	-	1.70%	0.20%	3.30%
LC10	*BRCA2*	p.K2833N	c.G8499C	-	-	-	4.60%
	CREBBP	p.Q931E	c.C2791G	-	-	-	1.40%
	EGFR	p.L858R	c.T2573G	1.42%	35.88%	2.66%	46.60%
	EPAS1	p.S474T	c.G1421C	0.40%	-	-	1.30%
	PGR	p.D697H	c.G2089C	-	-	-	8.60%
	PIK3R1	NA	HMGN1&WRB-PIK3R1	-	33.30%	-	19.80%
	RET	p.R820S	c.C2458A	1.43%	18.02%	-	15.50%
	SMAD4	p.W99C	c.G297T	0.30%	25.18%	-	2.40%
LC11	*CDKN1C*	p.V37L	c.G109T	-	0.20%	-	6.30%
	KDM5A	p.D1356N	c.G4066A	-	-	-	4.10%

	RNF43	p.P581L	c.C1742T	-	0.10%	-	4.70%
	CDK12	NA	SLCO5A1-CDK12	-	-	-	19.60%
LC12	*APC*	p.Q1242X	c.C3724T	7.56%	28.32%	-	1.10%
	SMAD4	p.R361C	c.C1081T	10.41%	50.16%	-	0.60%
LC13	*EGFR*	p.G719S	c.G2155A	2.10%	10.00%	0.50%	31.90%
	EGFR	p.L861R	c.T2582G	3.00%	9.70%	0.20%	44.10%
	PCDH11X	p.M520I	c.G1560A	0.54%	1.83%	-	-
	PIK3CA	p.D352Y	c.G1054T	0.30%	2.20%	-	-
	PIK3R2	p.G103E	c.G308A	5.20%	3.80%	1.20%	-
	TP53	p.G199E	c.G596A	1.70%	10.60%	-	30.70%
LC14	*ALK*	NA	EML4-ALK	-	2.70%	0.30%	4.20%
	BRCA2	NA	c.A68-6T	1.00%	-	-	1.40%
	EP300	p.S952X	c.C2855G	-	1.00%	1.00%	8.40%
	SBDS	p.R19Q	NA	-	-	1.18%	8.30%
LC15	*EGFR*	p.L858R	c.T2573G	-	9.31%	2.62%	25.90%
	HNF1A	p.Y218C	c.A653G	2.89%	1.29%	-	-
	PALB2	p.Q692L	c.A2075T	-	5.57%	1.52%	9.70%
	PDCD1LG2	p.T136K	c.C407A	-	6.15%	1.79%	13.30%
LC16	*EGFR*	p.L858R	c.T2573G	-	12.60%	7.13%	17.70%
	NSD1	p.E243X	c.G727T	-	4.70%	6.64%	2.20%
	GRIN2A	p.C456Y	c.G1367A	-	5.20%	2.64%	-
	KMT2B	p.R1777W	c.C5329T	-	1.30%	2.28%	-
	CHEK2	p.C420W	c.C1260G	0.70%	-	-	-
LC17	*FAT1*	p.P264fs	c.792_810delATCAGAACTGGACAGGGAC	21.69%	36.92%	-	41.00%
	TP53	p.K120X	c.A358T	25.58%	50.38%	-	36.00%
	RB1	p.G89X	c.G265T	20.93%	42.44%	-	37.00%
	GATA3	p.M439V	c.A1315G	24.00%	38.25%	-	34.50%

	MAP3K1	p.G565R	c.G1693A	21.61%	30.58%	-	26.40%
	KDR	p.S1231R	c.C3693A	21.14%	30.35%	-	27.70%
	FLT4	p.P1137T	c.C3409A	17.95%	30.20%	-	29.10%
	FLT1	p.Y125H	c.T373C	42.44%	55.90%	-	43.10%
LC18	*EGFR*	p.745_750del	c.2235_2249delGGAATTAAGAGAAGC	-	1.17%	-	32.10%
	SMARCA4	p.E514X	c.G1540T	-	0.49%	-	7.50%
	TP53	p.G245D	c.G734A	-	1.17%	-	18.20%
LC19	*CTNNB1*	p.S37C	c.C110G	-	-	-	3.00%
	EGFR	p.746_750del	c.2236_2250delGAATTAAGAGAAGCA	0.20%	0.50%	2.00%	7.00%
	SMAD4	p.C127X	c.T381A	-	-	-	4.00%
	SMAD4	p.Q449X	c.C1345T	-	0.50%	1.00%	-
LC20	*AKT2*	p.V90L	c.G268T	-	-	-	14.70%
	EGFR	p.A755A	c.C2265G	-	3.30%	1.50%	17.80%
	EGFR	p.E709K	c.G2125A	-	4.20%	3.00%	19.00%
	EGFR	p.LREATSP747delinsS	c.2240_2257delTAAGAGAAGCAACATCTC	-	3.30%	1.50%	17.80%
	FLT4	p.G63E	c.G188A	0.10%	5.40%	2.60%	12.10%
	KMT2B	p.P2271T	c.C6811A	-	-	-	4.10%
	SMARCA4	p.P1180A	c.C3538G	-	2.80%	1.60%	-
	TGFBR2	p.Q29X	c.C85T	-	-	-	2.30%
LC21	*EGFR*	p.M766delinsMASV	c.2296_2297insTGGCCAGCG	2.70%	38.33%	35.93%	25.00%
	GRIN2A	p.L566H	c.T1697A	3.32%	2.40%	0.70%	-
	KDM5A	p.I1226F	c.A3676T	-	-	-	3.60%
	TP53	p.S241T	c.T721A	1.30%	39.71%	34.50%	23.30%

LC22	*AMER1*	p.E396Q	c.G1186C	0.10%	9.96%	19.35%	-
	DDR2	p.R135H	c.G404A	0.22%	16.70%	21.42%	-
	EGFR	p.L858R	c.T2573G	0.10%	9.48%	15.27%	7.90%
	EPAS1	p.S797N	c.G2390A	-	2.11%	3.31%	-
	PIK3R2	NA	c.1291-2_1306delAGGACCAGATTGTCAAGG	-	-	-	5.40%
	PKD1	p.A3587fs	c.10761delG	0.10%	11.58%	17.47%	7.30%
	TP53	NA	c.A994-2G	0.10%	27.97%	41.53%	14.00%
LC23	*ALK*	NA	EML4-ALK	0.50%	1.40%	0.20%	10.00%
	STAT3	p.P330R	c.C989G	-	4.90%	-	-
LC24	*APC*	p.E2737D	c.G8211C	-	-	-	1.20%
	BRIP1	p.R965K	c.G2894A	20.00%	0.10%	-	-
	EGFR	p.L858R	c.T2573G	7.35%	29.68%	20.64%	16.40%
	MTOR	p.V715M	c.G2143A	7.26%	46.97%	17.21%	12.80%
	RECQL4	p.F201L	c.T603G	-	-	-	1.30%
	TP53	p.E204X	c.G610T	8.62%	62.27%	20.64%	13.50%
	TSHR	p.H484N	c.C1450A	3.25%	18.64%	9.33%	5.70%
LC25	*ABCB1*	NA	c.G2786+1A	-	14.29%	1.56%	13.90%
	ARAF	p.M355I	c.G1065T	-	12.53%	-	21.50%
	KRAS	p.G12D	c.G35A	-	16.18%	11.80%	22.80%
	NKX2-1	p.L347fs	c.1040_1044delTGTCC	-	16.49%	2.40%	28.00%
	RECQL4	p.P821P	c.A2463C	-	-	3.79%	-
	SMAD4	p.R361H	c.G1082A	-	1.01%	2.57%	0.50%
	TP53	p.V216M	c.G646A	-	11.73%	3.06%	23.40%
LC26	*ATRX*	p.D2352E	c.T7056A	-	1.15%	-	-
	ATRX	p.I2353R	c.T7058G	-	1.10%	-	-
	CBLB	p.Q193X	c.C577T	-	-	-	2.40%

	CREBBP	p.S78C	c.A232T	-	-	-	4.10%
	EGFR	p.746_750del	c.2236_2250delGAATTAAGAGAAGCA	0.80%	58.30%	47.90%	44.00%
	FLT4	p.T168M	c.C503T	-	1.27%	1.93%	-
	HGF	p.R134C	c.C400T	0.60%	25.96%	20.37%	-
	SMARCA4	p.P218Q	c.C653A	0.10%	26.42%	17.01%	-
	TP53	p.E258K	c.G772A	0.20%	81.57%	41.53%	33.00%
LC27	*AMER1*	p.P754S	c.C2260T	11.38%	67.51%	-	53.60%
	BIRC3	p.D466Y	c.G1396T	3.91%	23.36%	-	24.20%
	ERBB2	p.R103X	c.C307T	-	-	-	23.90%
	FANCD2	p.G583C	c.G1747T	4.53%	42.21%	-	25.10%
	FGFR4	p.P300S	c.C898T	9.09%	63.06%	0.59%	44.10%
	KMT2B	p.Q2353H	c.G7059T	3.88%	23.48%	-	25.90%
	NOTCH2	p.E1488Q	c.G4462C	-	-	-	25.50%
	PIK3CA	p.H1047R	c.A3140G	-	-	-	25.20%
	RB1	p.563fs	c.1689_1690insCT	4.17%	38.30%	-	32.40%
	TGFBR2	p.G399R	c.G1195C	6.24%	25.92%	-	24.40%
	TP53	NA	c.A920-2T	8.57%	68.07%	-	39.60%
LC28	*EGFR*	p.745_750del	c.2235_2249del	6.00%	36.00%	37.00%	36.00%
	MLH1	p.D668fs	c.2003_2010del	-	1.30%	1.20%	-
	STAG2	p.H608P	c.A1823C	-	19.32%	14.04%	8.50%
LC29	*EGFR*	p.L858R	c.T2573G	16.02%	14.85%	15.93%	4.40%
	NTRK1	p.P366L	c.C1097T	1.76%	-	-	-
	RB1	p.D332N	c.G994A	6.16%	-	-	-
	RICTOR	p.E677V	c.A2030T	12.23%	9.44%	8.47%	2.70%
	STAG2	p.H832L	c.A2495T	17.90%	16.05%	19.75%	9.90%
LC30	*NKX2-4*	p.K234N	c.G702C	-	8.00%	17.00%	-

	RET	NA	KIF5B-RET	1.00%	3.00%	5.00%	3.00%
	SETD2	p.K347R	c.A1040G	2.00%	-	-	7.00%
	SETD2	p.R349fs	c.1045delA	2.00%	-	-	7.00%
	SMAD4	p.R361C	c.C1081T	-	-	2.00%	-
	TGFBR2	p.L529R	c.T1586G	-	8.00%	26.00%	-
	TP53	p.Y234C	c.A701G	2.00%	1.00%	1.00%	1.00%
LC31	*ARID1A*	p.L2174R	c.T6521G	-	96.09%	63.70%	NA
	EGFR	p.745_750del	c.2235_2249delGGAATTAAGAGAAGC	0.20%	61.29%	49.74%	NA
	EGFR	p.T790M	c.C2369T	0.10%	10.44%	8.84%	NA
	IL7R	p.V253L	c.G757C	-	4.46%	7.65%	NA
	JAK3	p.359_360del	c.1076_1078delTCT	-	22.51%	16.17%	NA
	NOTCH1	p.G35E	c.G104A	-	26.26%	16.77%	NA
	PDGFRA	p.R822H	c.G2465A	-	14.18%	5.65%	NA
	TP53	p.R248W	c.C742T	0.10%	95.59%	66.62%	NA
LC32	*EGFR*	p.745_750del	c.2235_2249delGGAATTAAGAGAAGC	6.80%	40.71%	23.79%	NA
	TP53	p.N263fs	c.788_792delATCTA	5.20%	22.00%	12.09%	NA
	ALK	p.P74T	c.C220A	1.90%	18.40%	8.70%	NA
	TNFSF11	p.P34S	c.C100T	1.80%	8.30%	5.63%	NA
	CDKN2B	NA	MTAP&CDKN2A-CDKN2B	1.30%	16.10%	7.10%	NA
	DAXX	p.C664R	c.T1990C	-	1.20%	0.37%	NA
	MAP2K2	p.R193X	c.C577T	-	1.10%	0.57%	NA
LC33	*MET*	NA	c.2942-5_2942-9delinsAAAA	0.67%	44.60%	0.40%	NA

	NRAS	p.G13D	c.G38A	0.10%	1.96%	-	NA
	TP53	p.C229fs	c.686_687delGT	1.08%	38.16%	-	NA
	PGR	p.227fs	c.681_682insT	0.70%	36.21%	0.40%	NA
	SETD2	NA	SETD2-CCDC12&PTH1R	-	3.40%	-	NA
LC34	*EGFR*	p.745_750del	c.2235_2249delGGAATTAAGAGAAGC	0.50%	36.00%	47.00%	NA
	TP53	p.R213X	c.C637T	1.00%	60.00%	36.00%	NA
	TOP2A	p.P1400fs	c.4199delC	-	6.00%	9.00%	NA
	EP300	p.Q740fs	c.2219_2234delCTCAACCTGGAGCTCT	-	7.00%	8.00%	NA
	CDK12	NA	PENK&LINC00968-CDK12	-	7.00%	6.00%	NA
LC35	*APC*	p.L1509fs	c.4526_4527delTG	14.00%	-	-	NA
	EGFR	p.745_750del	c.2235_2249delGGAATTAAGAGAAGC	10.00%	4.00%	-	NA
	SMAD4	p.Y95H	c.T283C	11.00%	4.00%	-	NA
LC36	*EGFR*	p.L833V	c.T2497G	-	1.00%	-	NA
	EGFR	p.L858R	c.T2573G	-	1.00%	-	NA
LC37	*EPHA3*	p.E686K	c.G2056A	0.40%	2.45%	-	NA
	KRAS	p.G12D	c.G35A	1.00%	24.00%	1.00%	NA
	NKX2-1	p.T242fs	c.726_730delCGGGT	3.00%	20.00%	1.00%	NA
	TSC2	p.Q370X	c.C1108T	2.00%	21.00%	1.00%	NA
LC38	*FLT4*	p.G991E	c.G2972A	-	8.85%	7.18%	NA
	TP53	p.R248Q	c.G743A	0.17%	7.96%	6.78%	NA

	EGFR	p.A750P	c.G2248C	0.21%	14.52%	6.25%	NA
	EGFR	p.746_748del	c.2236_2244delGAATTAAGA	0.21%	13.37%	5.93%	NA
	BRIP1	p.E1230Q	c.G3688C	-	6.52%	6.40%	NA
	SMARCB1	p.R370G	c.A1108G	0.20%	13.33%	3.37%	NA
LC39	*ARID1A*	p.Q1614X	c.C4840T	0.30%	9.00%	1.00%	NA
	KRAS	p.G12C	c.G34T	-	17.00%	0.50%	NA
	TP53	p.R158L	c.G473T	-	13.00%	1.00%	NA
	BRIP1	p.Y822C	c.A2465G	-	10.00%	1.00%	NA
	FAT1	p.D2891Y	c.G8671T	-	7.00%	1.00%	NA
	MSH6	p.R1182K	c.G3545A	-	1.45%	-	NA
	ARID2	p.G882fs	c.2644delG	-	25.00%	1.00%	NA
	CHEK2	NA	KIAA1671-CHEK2	-	-	6.00%	NA
LC40	*EGFR*	p.ELREATS746delinsA	c.2237_2254delAATTAAGAGAAGCAACAT	2.10%	73.38%	82.61%	NA
	EGFR	p.S752F	c.C2255T	2.05%	73.40%	82.60%	NA
	BRAF	p.E586K	c.G1756A	0.10%	21.88%	23.37%	NA
	KRAS	p.G12F	c.GG34_35TT	0.20%	26.18%	33.50%	NA
	RET	p.N448S	c.A1343G	0.40%	25.84%	40.01%	NA
	MTOR	p.K980R	c.A2939G	-	13.22%	28.21%	NA
	NTRK1	p.D776N	c.G2326A	0.50%	18.98%	29.44%	NA
	RICTOR	p.E1123K	c.G3367A	0.10%	6.12%	5.78%	NA
	SUFU	p.G294D	c.G881A	0.20%	1.29%	-	NA
LC41	*CDKN1B*	p.E86X	c.G256T	1.17%	3.00%	14.81%	NA
	CDKN2A	p.Y129X	c.C387A	1.03%	3.00%	13.85%	NA
	CDKN2A	p.R58X	c.C172T	1.00%	3.00%	15.77%	NA
	CYLD	p.A211V	c.C632T	1.96%	4.51%	15.77%	NA

	ERBB2	p.771insAYVM	c.2310_2311insGCATACGTGATG	1.00%	3.00%	28.12%	NA
	ESR1	p.A318V	c.C953T	1.73%	4.11%	11.23%	NA
	FLT1	p.V587I	c.G1759A	-	-	1.37%	NA
	MTOR	p.L2037S	c.T6110C	-	0.99%	2.79%	NA
	NSD1	p.R2601S	c.G7803C	2.11%	3.56%	18.46%	NA
LC42	*BRCA2*	p.S2691F	c.C8072T	7.45%	0.94%	-	NA
	BRCA2	p.S2704F	c.C8111T	1.65%	0.40%	-	NA
	BRCA2	p.L2753F	c.C8257T	1.62%	0.71%	-	NA
	BRCA2	p.H2754Y	c.C8260T	1.57%	0.48%	-	NA
	BRCA2	p.P2735S	c.C8203T	1.43%	0.86%	-	NA
	BRCA2	p.S2701C	c.C8102G	3.06%	0.54%	-	NA
	CDK6	p.D242H	c.G724C	2.78%	0.32%	-	NA
	CDKN1C	p.Q226H	c.G678C	2.69%	-	-	NA
	CREBBP	p.E1035Q	c.G3103C	2.56%	0.47%	-	NA
	EGFR	p.L858R	c.T2573G	2.52%	28.44%	15.56%	NA
	PTEN	NA	c.T209+2A	2.78%	0.93%	-	NA
	PTEN	NA	c.209_209+1delTG	2.78%	0.93%	-	NA
	MET	p.L1213F	c.C3637T	1.44%	-	-	NA
	RAC1	p.A172V	c.C515T	0.86%	-	-	NA
	APC	p.G2836V	c.G8507T	0.74%	16.95%	7.64%	NA
	CTNNB1	p.E738K	c.G2212A	0.58%	10.55%	6.00%	NA
	NPM1	p.L18V	c.C52G	0.59%	7.96%	4.38%	NA
LC43	*EGFR*	p.L858R	c.T2573G	0.50%	38.00%	5.00%	NA
	CDK12	p.L554F	c.G1662T	0.80%	23.00%	3.00%	NA
	THADA	p.T668I	c.C2003T	-	21.00%	2.00%	NA
LC44	*EGFR*	p.G719A	c.G2156C	-	41.00%	17.00%	NA
	EGFR	p.R776H	c.G2327A	-	43.00%	19.00%	NA

	TP53	p.M246L	c.A736T	-	6.43%	0.84%	NA
	RICTOR	p.R380C	c.C1138T	-	4.41%	2.45%	NA
LC45	*EGFR*	p.745_750del	c.2235_2249delGGAATTAAGAGAAGC	32.41%	37.00%	20.00%	NA
	EGFR	p.T790M	c.C2369T	22.00%	24.00%	15.00%	NA
	TP53	p.H179L	c.A536T	17.00%	26.00%	11.00%	NA
LC46	*TEK*	p.Y1080C	c.A3239G	0.88%	0.75%	0.52%	NA
	ROS1	p.D1253H	c.G3757C	0.20%	16.04%	1.65%	NA
	ROS1	p.D1255N	c.G3763A	0.20%	16.04%	1.56%	NA
	SETD2	p.Q1272X	c.C3814T	-	9.65%	0.37%	NA
	ROS1	NA	SLC34A2-ROS1	-	4.11%	1.05%	NA
	AXIN2	p.S52F	c.C155T	0.24%	32.64%	3.18%	NA
	EPHA3	p.I45F	c.A133T	0.10%	16.47%	0.85%	NA
	ERBB4	p.D871H	c.G2611C	-	17.89%	2.00%	NA
	EXT1	p.D654N	c.G1960A	0.19%	9.01%	0.87%	NA
	FAT1	p.T1338I	c.C4013T	-	8.61%	0.98%	NA
	MYD88	p.E104Q	c.G310C	0.10%	12.82%	0.61%	NA
	NTRK1	p.P392L	c.C1175T	-	9.67%	1.08%	NA
	PKHD1	p.S7C	c.C20G	-	8.71%	-	NA
	SLC34A2	NA	ANAPC4&SLC34A2-SLC34A2:EXON5	0.10%	5.34%	0.67%	NA
LC47	*ERBB2*	p.771insAYVM	c.2310_2311insGCATACGTGATG	0.80%	13.00%	0.60%	NA
	TP53	p.C277F	c.G830T	1.21%	17.98%	1.00%	NA
	ABCB1	p.R588H	c.G1763A	1.00%	4.52%	0.50%	NA
	JAK3	p.G313R	c.G937A	-	1.00%	0.60%	NA
	MED12	p.R753W	c.C2257T	1.52%	22.73%	2.60%	NA

	MTOR	p.D2135N	c.G6403A	0.50%	4.36%	-	NA
	RB1	p.469fs	c.1407_1408insATTCAAA	-	12.80%	-	NA
	VEGFA	p.D3fs	c.7_10delGACA	0.20%	0.85%	-	NA
LC48	*EGFR*	p.746_750del	c.2236_2250delGAATTAAGAGAAGCA	0.40%	1.00%	1.00%	NA
	EGFR	p.T790M	c.C2369T	-	0.40%	-	NA
LC49	*EGFR*	p.S768I	c.G2303T	-	32.89%	13.41%	NA
	EGFR	p.G719A	c.G2156C	-	32.52%	14.26%	NA
	MDM4	p.C10S	c.G29C	-	11.45%	7.09%	NA
	CREBBP	p.G2019A	c.G6056C	0.10%	1.94%	1.40%	NA
	KMT2B	p.286_289del	c.857_865delGCCGTGGAG	-	5.47%	-	NA
	TP53	p.Y205C	c.A614G	-	1.00%	2.18%	NA
LC50	*EGFR*	p.745_750del	c.2235_2249delGGAATTAAGAGAAGC	5.07%	0.20%	0.20%	NA
	CTNNB1	p.S45F	c.C134T	-	2.00%	0.10%	NA
	TP53	p.Y220C	c.A659G	0.20%	1.00%	-	NA
	BTK	p.Q260R	c.A779G	-	1.17%	-	NA
	FANCD2	p.L966H	c.T2897A	-	1.26%	-	NA
	IL7R	p.A432D	c.C1295A	1.33%	-	-	NA
	POLH	p.P312H	c.C935A	1.33%	-	0.60%	NA
LC51	*EGFR*	p.L858R	c.T2573G	0.40%	22.40%	34.20%	NA
	EPHA2	NA	c.G2325+1A	1.60%	0.90%	0.60%	NA
	MAP3K1	p.T949A	c.A2845G	1.80%	-	-	NA
	CTNNB1	p.S37F	c.C110T	-	0.80%	1.70%	NA
	FANCA	NA	c.G1566+1T	-	8.50%	12.10%	NA
	TP53	p.R249S	c.G747T	-	10.90%	16.10%	NA

	TP53	p.P250S	c.C748T	-	10.90%	16.10%	NA
	TP53	p.P190S	c.C568T	-	16.70%	17.90%	NA
	CDC73	p.Q490E	c.C1468G	-	0.40%	1.80%	NA
	GATA6	p.P544L	c.C1631T	-	8.60%	8.40%	NA
LC52	*EGFR*	p.L861Q	c.T2582A	47.56%	50.04%	32.00%	NA
	EGFR	p.T790M	c.C2369T	36.00%	-	-	NA
	TP53	p.P151A	c.C451G	6.00%	24.64%	15.00%	NA
	PTEN	p.H93R	c.A278G	8.00%	11.00%	13.00%	NA
	APC	p.I605fs	c.1814_1815delAT	0.10%	5.04%	4.00%	NA
	PIK3CA	p.E545K	c.G1633A	-	2.93%	1.00%	NA
	ARID1A	p.S1134C	c.C3401G	-	2.27%	4.09%	NA
	ATRX	p.R1401Q	c.G4202A	-	1.73%	3.26%	NA
	ERBB4	p.E1199K	c.G3595A	-	-	1.00%	NA
	FANCA	p.L910R	c.T2729G	5.61%	17.34%	32.14%	NA
	FLCN	p.R179W	c.C535T	-	-	1.88%	NA
	GRIN2A	p.T626A	c.A1876G	1.44%	0.53%	2.28%	NA
LC53	*EGFR*	p.746_750del	c.2236_2250delGAATTAAGAGAAGCA	0.80%	60.00%	2.00%	NA
	TP53	NA	c.A920-2T	0.10%	40.00%	1.00%	NA
	BARD1	p.V614F	c.G1840T	-	1.35%	-	NA
	CBL	p.S899F	c.C2696T	-	11.30%	-	NA
	GSTT1	p.S11A	c.T31G	-	38.12%	-	NA
	KMT2A	p.L642R	c.T1925G	-	20.12%	0.57%	NA
	PTCH1	p.R441C	c.C1321T	-	32.30%	0.83%	NA
LC54	*KRAS*	p.G12C	c.G34T	0.30%	52.00%	25.00%	NA
	TP53	p.C135F	c.G404T	0.10%	46.00%	20.00%	NA
	CDK12	p.I147L	c.A439T	0.30%	20.00%	12.00%	NA
	MYD88	p.L265P	c.T794C	-	-	2.05%	NA

LC55	*TP53*	p.V272L	c.G814T	2.00%	80.00%	37.00%	NA
	ERBB2	p.771insAYVM	c.2310_2311insGCATACGTGATG	2.00%	64.00%	28.00%	NA
	NF2	p.A373V	c.C1118T	1.00%	34.00%	16.00%	NA
	ROS1	p.G272C	c.G814T	2.00%	1.00%	1.00%	NA
LC56	*TP53*	p.Q136E	c.C406G	11.00%	5.00%	0.30%	NA
LC57	*EGFR*	p.709_710del	c.2127_2129del	2.00%	81.00%	36.00%	NA
	TP53	p.R273L	c.G818T	1.00%	41.00%	5.00%	NA
	AR	p.R753X	c.C2257T	2.00%	-	-	NA
LC58	*TP53*	p.R273Q	c.GT818_819AG	2.00%	7.00%	1.00%	NA
	EGFR	p.L858R	c.T2573G	1.00%	7.00%	2.00%	NA
	PIK3CA	p.E726K	c.G2176A	1.00%	5.00%	-	NA
	PIK3CA	p.E542K	c.G1624A	1.00%	3.00%	-	NA
	PDGFRA	p.V859M	c.G2575A	0.40%	3.00%	1.00%	NA
LC59	*BRCA2*	p.S2372X	c.C7115G	1.53%	72.41%	7.55%	NA
	EGFR	p.745_750del	c.2235_2249delGGAATTAAGAGAAGC	1.10%	41.80%	5.90%	NA
	FGFR1	p.V14G	c.T41G	0.30%	14.42%	1.50%	NA
	PTEN	p.R130Q	c.G389A	0.30%	41.09%	2.60%	NA
	TNFAIP3	p.L435fs	c.1303_1312delCTCGGGGCCT	0.30%	0.10%	2.00%	NA
	TP53	p.R282G	c.C844G	0.70%	60.40%	5.92%	NA
	TSC2	p.R413fs	c.1238_1245delGATGTGCG	0.10%	0.63%	-	NA
	PAX5	NA	MSC&EYA1-PAX5	-	0.80%	-	NA

LC60	*EGFR*	p.745_750del	c.2235_2249delGGAATTAAGAGAAGC	28.00%	10.00%	2.00%	NA
	IGF1R	p.R650W	c.C1948T	4.94%	1.03%	-	NA
	MYD88	p.V217F	c.G649T	0.26%	2.12%	3.01%	NA
LC61	*EGFR*	p.745_750del	c.2235_2249delGGAATTAAGAGAAGC	-	9.00%	4.00%	NA
LC62	*EGFR*	p.745_750del	c.2235_2249delGGAATTAAGAGAAGC	1.00%	9.00%	2.00%	NA
	TP53	NA	c.993+1delG	1.00%	14.00%	1.00%	NA
LC63	*EGFR*	p.747_750del	c.2239_2250delTTAAGAGAAGCA	0.30%	3.00%	12.00%	NA
	EGFR	p.E746V	c.AA2237_2238TT	0.30%	3.00%	12.00%	NA
	EGFR	p.T751A	c.A2251G	0.30%	3.00%	12.00%	NA
	EGFR	p.T790M	c.C2369T	0.20%	0.40%	3.00%	NA
	PKHD1	p.W656R	c.T1966C	1.00%	0.30%	0.20%	NA
	CHD4	p.P584A	c.C1750G	2.00%	1.00%	0.40%	NA
	ALK	p.Q1159X	c.C3475T	0.46%	1.34%	-	NA
	RUNX1	p.M371V	c.A1111G	0.17%	0.40%	1.11%	NA

MAF, mutant allele frequency; -, undetectable; NA, not available.
